# A data science-led strategy to assess the subnational burden of sepsis using official records: a longitudinal description and cross-sectional demonstration in Chile

**DOI:** 10.3389/fmed.2025.1671206

**Published:** 2026-01-12

**Authors:** Sebastian Gatica, Niranjan Kissoon

**Affiliations:** 1 Escuela de Tecnología Médica, Facultad de Salud, Universidad Santo Tomas, Santiago, Chile; 2 Centro de Investigación en Prevención y Cuidados de la Salud (+ SALUD), Facultad de Salud, Universidad Santo Tomas, Santiago, Chile; 3 Department of Pediatrics, University of British Columbia, Vancouver, BC, Canada

**Keywords:** burden of disease, data science, LMIC, public health, public policies, sepsis

## Abstract

Sepsis is a life-threatening organ dysfunction caused by an aberrant host response to an infecting pathogen. Several international efforts have been launched to address its staggering burden and escalating costs. A disconnection occurs when translating current global, regional, and national estimates into local and subnational quantifications of their burden. Given the level of completeness of civil registration and vital statistics in Chile, an opportunity arose to calculate, rather than estimate, the burden of sepsis by subnational administrative division. Thus, for the first time, a data science-driven strategy is presented to quantify sepsis-related incidence and mortality from official datasets in this country. Moreover, given the high-throughput potential of the analysis, areas where sepsis-related mortality exceeded its incidence were identified by administrative division, age group, and individual cause of death, and ranked by the magnitude of the excess. Thus, a strategy to guide the efficient deployment of public health resources based on subnational burden is presented. Implementation of such a strategy may represent the key to tackling sepsis with a local-to-global perspective, especially in low- and middle-income countries.

## Introduction

Sepsis is defined as a life-threatening organ dysfunction stemming from an aberrant host response to an infecting pathogen (1). It is estimated that roughly 1/5 of all global deaths are related to sepsis (2). With initial hospitalization costs rising (3) and a readmission rate of up to 16.4% (4), sepsis costs account for up to 15.85% of the national healthcare budget (5). Apart from the acute episode of sepsis, quality-adjusted life years (QALY) lost due to sepsis are estimated to be about 2/3 of the estimated QALY lost due to the COVID-19 pandemic at its peak, and productivity lost after sepsis is estimated to be 4–7 times higher than coronary heart disease or stroke in Europe (6). Efforts to quantify sepsis incidence and mortality to inform strategic allocation of public health resources have become a global endeavor (7). However, local official data are needed to chart and address the burden, implement prevention and quality improvement measures and programs, improve outcomes, and benchmark progress (8). Since public health policies are ultimately applied locally (i.e., on a county-by-county, state-by-state, and country-by-country basis), granular subnational analysis is the most suitable approach to tackle sepsis efficiently by allocating resources where they are most needed.

Chile provides an excellent example of serving as a model for quantifying sepsis from local to national levels, as its official civil registration and vital statistics systems for death registration and medically certified causes of death are 100% complete (9, 10). This offers a rare opportunity to demonstrate the value of our strategy for assessing the subnational burden of sepsis, not only in Latin America but across all member countries of the Organization for Economic Co-operation and Development (OECD). We believe that this granular approach provides a template to deploy public health resources efficiently, especially in areas with limited resources in low- and middle-income countries (LMICs), significantly contributing to the goals of the Global Sepsis Alliance (GSA) 2030 agenda for sepsis (11), the 2025 European Sepsis Alliance (ESA) call to action (12), the 2025–2029 Pan American Health Organization’s Strategy and Plan of Action to Decrease the Burden of Sepsis (13), the Sustainable Health Agenda for the Americas (14), and the 2017 World Health Organization’s sepsis resolution (7).

Therefore, the objective of this investigation is to report a data-science strategy to quantify sepsis-related incidence in Chile and to identify local areas and/or specific causes where sepsis-associated mortality exceeds that incidence. We hypothesize that such a strategy enables: (1) the quantification of the sepsis-related burden from official datasets by subnational administrative division and (2) the determination of the individual causes for which sepsis-related mortality exceeds its incidence.

## Materials and methods

**Figure 1:**
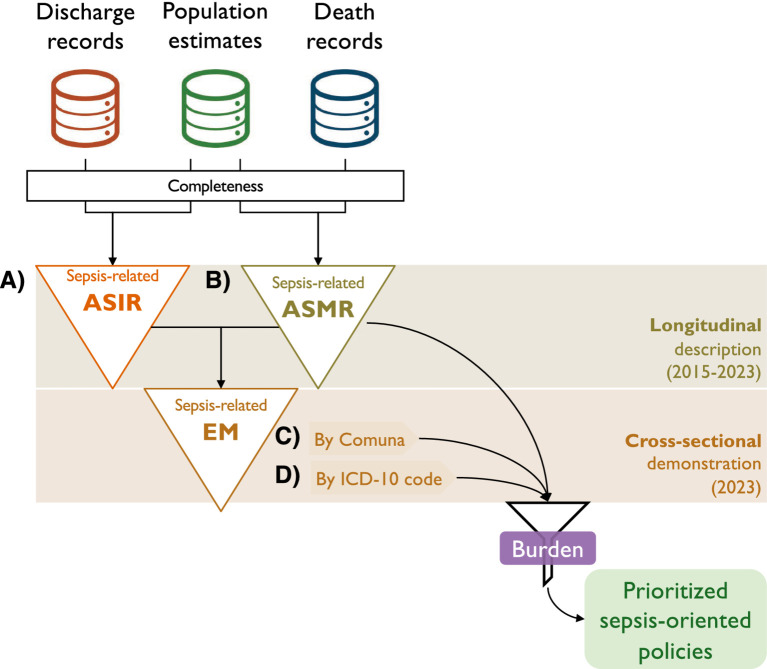
Graphic summary of the strategy to assess the subnational burden of sepsis-related cases and the specific objective of this study. Official datasets on hospital discharges, population estimates, and death records in Chile were accessed electronically and used as input for a retrospective analysis to assess the subnational burden of sepsis and identify the individual causes for which sepsis-related mortality exceeds its incidence. After assessing row completeness, crude sepsis-related incidence and mortality were calculated by year, sex, age type, age magnitude, Comuna, Region, and ICD-10 code. Then, age-standardized (2025 population matched by country, region, or Comuna) incidence **(A)**, ASIR and mortality **(B)**, ASMR were calculated. Then, ASIR and ASMR were integrated to determine sepsis-related excess of mortality (EM) by Comuna **(C)** and ICD-10 code **(D)**. Filtered this way, Comunas where sepsis-related mortality exceeded its incidence can be identified.

This report followed the Strengthening the Reporting of Observational Studies in Epidemiology (STROBE) checklist (15). Due to the retrospective design, written informed consent was waived for all subjects. A general outline of the strategy is presented in Figure 1. A longitudinal approach was used to describe trends in sepsis-related rates from 2015 to 2023. Because data availability was granular in the Comuna analyses, interpretability was preserved by adopting a cross-sectional approach, focusing on the latest available figure (2023) (Figure 1). Official datasets on hospital discharges, population estimates, and death records in Chile were accessed electronically and used as input for a retrospective cross-sectional demonstration of our strategy to assess the subnational burden of sepsis. Data were handled using R version 4.3.2. Death records (excluding fetal deaths) were retrieved from the Chilean Department of Health Statistics and Information (*Departamento de Estadísticas e Información de Salud*, DEIS) (16) for the years 2015–2023 as .csv datasets (Supplementary Figure 1). Administrative divisions of Chile comprise Regions (16 first-level divisions), Provinces (56 s-level divisions, not explored in this analysis), and Comunas (346 third-level divisions). Interpretability was preserved by excluding the variable sex from the region and Comuna analyses.

After extracting variables related to year of death, sex, type of age, age, Region, and Comuna of residency, and underlying cause of death as a single International Classification of Diseases (ICD)-10 code, a dataframe with 1,052,987 cases, comprising records from 2015 to 2023, was obtained and characterized for missing and undetermined content variable-wise (Supplementary Figure 1). Based on this root dataframe, successive dataframes were prepared according to the assessed administrative depth. Details about dataframe processing and filtering, as well as missing and undetermined content of each variable considered, are depicted in comprehensive detail in Supplementary Figure 1. Hospital discharges were also retrieved from Chilean DEIS for years 2015–2023 as .csv datasets (Supplementary Figure 2). After excluding cases of mortality and extracting variables related to year of discharge, sex, age, Region, and Comuna of residency, and primary cause of hospitalization as a single ICD-10 code, hospital discharges with an «alive» outcome were merged into a single 13,942,481-case dataframe, comprising records from 2015 to 2023 (Supplementary Figure 2). Based on this root dataframe, successive dataframes were prepared according to the assessed administrative depth. Details about dataframe processing and filtering, as well as missing and undetermined content of each variable considered, are depicted in comprehensive detail in Supplementary Figure 2. Finally, population figures were retrieved from the Chilean National Institute of Statistics (*Instituto Nacional de Estadísticas*, INE) (17) by region and Comuna for the years 2002–2035, compiled into a single .csv file.

To quantify bias from single-code analyses, death records were retrieved from the Brazilian Department of Information and Informatics of the Unified Health System (*Departamento de Informação e Informática do Sistema Único de Saúde*, DATASUS) (18) for years 2015–2023 as .dbc datasets (Supplementary Figure 3). After extracting variables related to the date of death, type of death (i.e., fetal or not), sex, age, and causes of death as multiple ICD-10 codes corresponding to lines A-D of the death certificate, a dataframe with 12,952,481 cases, comprising records from 2015 to 2023, was obtained. Based on this root dataframe, two dataframes were prepared using either a single-cause-of-death (SCODe) or a multiple-cause-of-death (MCODe) approach. Details about dataframe processing and filtering, as well as missing and undetermined content of each variable considered, are depicted in comprehensive detail in Supplementary Figure 3. Additionally, hospital discharges were also retrieved from Brazilian DATASUS for the years 2015–2023 as .csv datasets (Supplementary Figure 4). After excluding cases of mortality and extracting variables related to date of discharge, sex, type of age, age, state and Municipalidad of occurrence, and primary and secondary cause of hospitalization as multiple ICD-10 codes (multiple cause of discharge, MCODi), hospital discharges were merged into a single 167,046,431 cases root dataframe, comprising records from 2015 to 2023 (Supplementary Figure 4). Details about dataframe processing and filtering, as well as missing content of each variable considered, are depicted in comprehensive detail in Supplementary Figure 4. Finally, Brazilian population figures were also retrieved from DATASUS by state and Municipalidad for the years 2015–2023 as multiple .dbf files.

Randomness bias in missing data was assessed using a chi-square goodness-of-fit test in data frames with variable completeness <99.95%. Within the root dataframes for death, discharge, and population, age was expressed as both a continuous and a categorical variable (Supplementary Table 1). Therefore, to enable comparability across dataframes and to preserve interpretability, age was harmonized and recoded as a categorical variable of the following levels: <1, 1–19, 20–39, 40–59, and >60 years old. Variables related to ICD-10 codes were queried for structural integrity (e.g., cases including illegal or lowercase characters, missing the first-letter component, ending in a letter, etc.) and length (i.e., 3- or 4-character codes). Four-character codes with “X” as the fourth character were trimmed to three characters by removing this placeholder.

Sepsis was defined using ICD-10 codes listed in the report by Rudd et al. (2) (Supplementary Table 2). Limited by the availability of a single code per death or discharge record in Chilean datasets, an SCOD (i.e., SCODe and SCODi) approach was used, classifying sepsis-related cases explicitly [e.g., sepsis due to *Staphylococcus aureus* (A41.0)] or implicitly (i.e., by the presence of an infection or an organ dysfunction code listed in Supplementary Table 2; Supplementary Figure 5A). To quantify bias from SCOD analyses in Chilean datasets, representative external death and hospital discharge data (both from Brazilian DATASUS, as described above) under an MCOD form were processed to calculate a country-level correction ratio (λ) as the quotient between the MCOD age-standardized rate and the SCOD age-standardized rate. To this end, the Brazilian death root dataframe structure was inspected. Consistent with the death certificate layout, causes of death were found within variables LINHAA, LINHAB, LINHAC, and LINHAD, each containing a maximum of five ICD-10 codes in asterisk-demarcated slots (Supplementary Figure 5B). For the MCODe approach (Supplementary Figure 5A), sepsis cases were identified by explicit sepsis codes across all lines (Supplementary Figure 5B). Because Chilean DEIS provides only the underlying cause of death and the primary cause of hospitalization, the underlying cause of death and primary cause of hospitalization were used to identify sepsis cases under an SCOD approach (Supplementary Figure 5A) in the external data. Illustrative examples of sepsis identification under the Brazilian death root dataframe structure are presented in Supplementary Figure 5B.

Crude incidence and mortality rates for sepsis-related cases and other causes grouped by ICD chapter were calculated using the sum of corresponding deaths or discharges as the numerator and population figures (by corresponding administrative level) as the denominator. Crude rates were further adjusted by the age structure of the 2025 population to obtain age-standardized incidence rates (ASIR) and age-standardized mortality rates (ASMR), expressed per 100,000 population. Confidence intervals (CIs) were calculated for all administrative levels using a gamma/chi-squared approximation (19) with the R package epitools. Sensitivity in age-standardized rates was tested using a probabilistic sensitivity analysis (PSA, i.e., a Monte Carlo simulation), followed by a global sensitivity analysis assessed using Spearman’s rank correlation. Uncertainty was first inspected visually by highlighting the width of CIs at the Country and region levels, and by inspecting CIs in error bar charts at the Comuna level. Then, uncertainty was tested by assessing the variance of the difference between the region or Comuna age-standardized rates, followed by a Z-test. The ratio between ASMR and ASIR was calculated at the Comuna level by age and ICD-10 code. Ratios where ASMR > ASIR were called the «excess of mortality» (EM). Figure 1 summarizes both our strategy for assessing the subnational burden of sepsis-related cases and the specific objective.

## Results

### Dataset retrieval and completeness assessment

The retrieved intercensal population projections were 100% complete. In brief, the Chilean population was estimated to grow from 17,971,423 to 19,960,889 between 2015 and 2023. The specific composition of the population by age group from 2015 to 2023, including the standard population (the Chilean estimate for 2025) and its age-group-specific weights, is presented in Supplementary Table 3. The specific composition of the population by Region, Comuna, and age group from 2015 to 2023, including the standard population (the Chilean estimate for 2025) and its age-group-specific weights, is presented in Supplementary Table 4.

Death record input consisted of two .csv datasets covering years 2015–2021 and 2022–2023 (Supplementary Figure 1). After variable extraction and merging, the pooled input was 1,052,987 rows. From this root dataframe, a country-level dataframe of 1,052,815 rows was obtained by excluding variables Comuna and Region, as well as not available (NA) (maximum 0.001%) and undetermined (maximum 0.014%) content from the remaining variables (Supplementary Figure 1). From the root dataframe, a region level dataframe of 91,531 rows was obtained by excluding cases unrelated to sepsis and their NA (maximum 0.002%) and undetermined (maximum 0.004%) content, and a Comuna level dataframe of 14,034 rows was obtained by excluding variable Region, cases from 2015 to 2022, and the undetermined (maximum 0.014%) content (Supplementary Figure 1).

Hospital discharge record input consisted of nine .csv datasets covering the years 2015–2023 individually (Supplementary Figure 2). Variables were extracted from discharges with an «alive» outcome and merged into a single 13,942,481 row pooled input root dataframe. From this root dataframe, a country-level dataframe with 13,510,829 rows was obtained by excluding the variables Comuna and Region, as well as NA values (maximum 3.095%) and undetermined values (maximum 0.001%) from the remaining variables (Supplementary Figure 2). From the root dataframe, a region dataframe of 2,109,746 rows was obtained by excluding variable Comuna, cases unrelated to sepsis, and the NA (maximum 3.056%) and undetermined (maximum 0.083%) content, and a Comuna level dataframe of 240,521 rows was obtained by excluding variable Region, cases from 2015 to 2022, and undetermined (maximum 0.74%) content (Supplementary Figure 2).

The retrieved Brazilian intercensal population projections were 100% complete. In brief, the Brazilian population was estimated to grow from 202,403,642 to 211,695,158 between 2015 and 2023. The specific composition of the population by age group from 2015 to 2023, including the standard population (the Brazilian estimate for 2024, the latest year available) and its age-group-specific weights, is presented in Supplementary Table 5. Brazilian death record input consisted of 9 .dbc datasets, each covering the years 2015–2023 (Supplementary Figure 3). After variable extraction and merging, the pooled input was 12,952,481 rows. From this root dataframe, sepsis-related cases were identified with either an MCODe or SCODe approach (Supplementary Figure 5). For the MCODe approach, a 2,911,592-row MCODe dataframe was obtained after excluding cases unrelated to sepsis and their undetermined content (maximum 0.0358%) (MCODe dataframe in Supplementary Figure 3). For the SCODe approach, a 2,185,762-row SCODe dataframe was obtained after excluding cases unrelated to sepsis and their undetermined content (maximum 0.0679%) (SCODe dataframe in Supplementary Figure 3). Brazilian hospital discharge record input consisted of nine .dbc datasets, each covering the years 2015–2023 (Supplementary Figure 4). Variables were extracted from discharges with an «alive» outcome and merged into a single 167,046,431-row pooled input root dataframe. While NA content in this root dataframe was 13.29% per variable, the critical data on primary and secondary causes of hospitalization had 41.2 and 97.2% NA content, respectively (38.9 and 98.2% in 2023).

### Sepsis-related incidence and mortality

**Figure 2:**
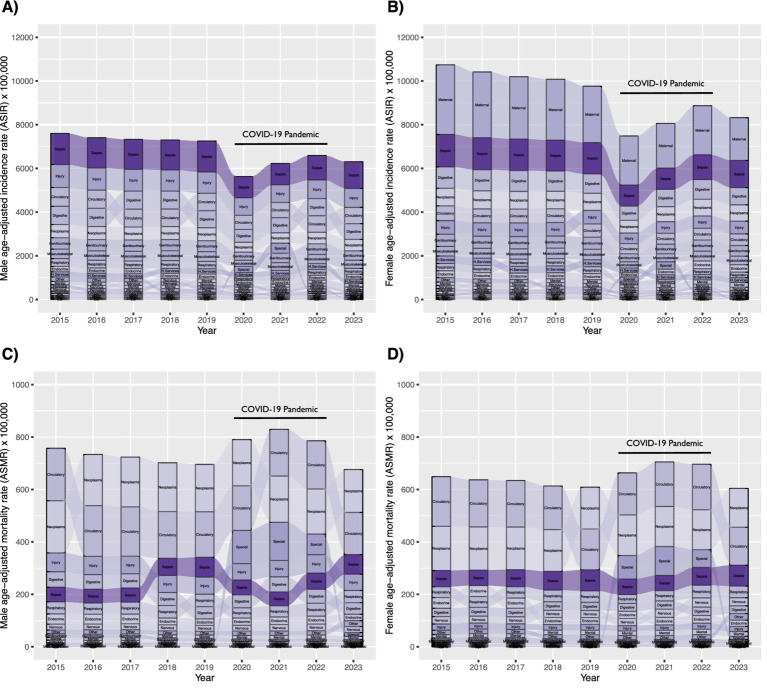
Ranking and progression of sepsis-related age-standardized incidence rate (ASIR) **(A)**, males; **(B)**, females and age-standardized mortality rate (ASMR) **(C)**, males; **(D)**, females), and 22 ICD chapters in Chile from 2015 to 2023.

**Figure 3:**
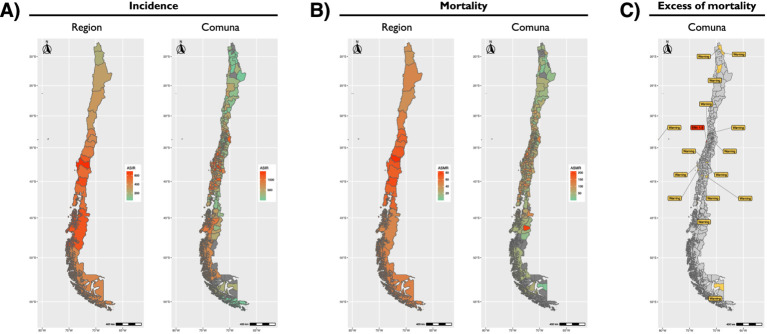
Geographic distribution of sepsis-related age-standardized incidence rate (ASIR) **(A)**, age-standardized mortality rate (ASMR) **(B)**, and excess of mortality (EM) **(C)**, during 2023. An improvement in resolution is exemplified by placing results by region next to results by Comuna.

Sepsis-related ASIR calculated from official Chilean records ranked consistently as the first cause of hospital discharge from 2015 to 2023 among males (Figure 2A). Females exhibited a similar pattern and magnitude for sepsis-related ASIR, except it ranked consistently as the second cause of hospital discharge (after causes related to pregnancy, childbirth, and the puerperium) during the same period (Figure 2B). The age group with the highest ASIR was>60 years old, although there was no significant dominance across the years studied (Supplementary Figures 6A,B). At the Country level, a negative slope in ASIR progression was observed between 2015 and 2023, with narrow CIs (Supplementary Figure 7A and Supplementary Table 6). A negative slope was also observed in all Regions, except for Coquimbo and, prominently, Arica y Parinacota, where a positive trend was observed between 2015 and 2023 (Supplementary Figure 8A and Supplementary Table 7). Comuna analysis of ASIR for the latest year available revealed a heterogeneous landscape (Supplementary Figure 9A and Supplementary Table 8), with greater magnitudes observed in Comunas around the center of the country (Figure 3A) and in the >60-year-old age group (Supplementary Figure 10).

Sepsis-related ASMR calculated from official Chilean records ranked between positions 6th and 3rd during 2015–2022 and ranked as the third cause of death during 2023, both for males (Figure 2C) and females (Figure 2D). The age group with the highest ASMR was>60 years old, with clear dominance across the years studied (Supplementary Figures 6C,D). At the Country level, a positive slope in ASMR progression was observed between 2015 and 2023, with narrow confidence intervals (CI) (Supplementary Figure 7B and Supplementary Table 9). A positive slope was also observed in all Regions between 2015 and 2023 (Supplementary Figure 8B and Supplementary Table 10). Comuna analysis of ASMR for the latest year revealed a heterogeneous landscape (Supplementary Figure 9B and Supplementary Table 11), with greater magnitudes observed in Comunas near the center of the country (Figure 3B) and in the >60-year-old age group (Supplementary Figure 10). Of note, the Comuna with the second-highest ASMR (Río Ibáñez) is located in a remote area with limited connectivity to the rest of the country.

### Sepsis-related excess of mortality by Comuna

The ratio between ASMR and ASIR was calculated at the Comuna level by age and code. Ratios in which ASMR > ASIR were designated «excess of mortality» (EM). Sepsis-related EM allows a rapid identification of Comunas where sepsis is not being contained. As such, only one Comuna (Navidad) was found to have an EM > 1, with ASMR of 41.6 and ASIR of 27.8 per 100,000 population (Figure 3C; Supplementary Figure 10, and Supplementary Table 12). Comunas where ASMR>1 and ASIR = 0 were attributed a warning label for the mathematical limitation to quantify the magnitude of EM (Figure 3C). These 15 Comunas with an EM warning were Colchane, María Elena, Juan Fernández, Quintero, Algarrobo, Codegua, Lebu, Tirúa, Tucapel, Gorbea, Pucón, Queilén, Porvenir, Putre, and Treguaco.

### Sepsis-related EM by cause of death

**Figure 4:**
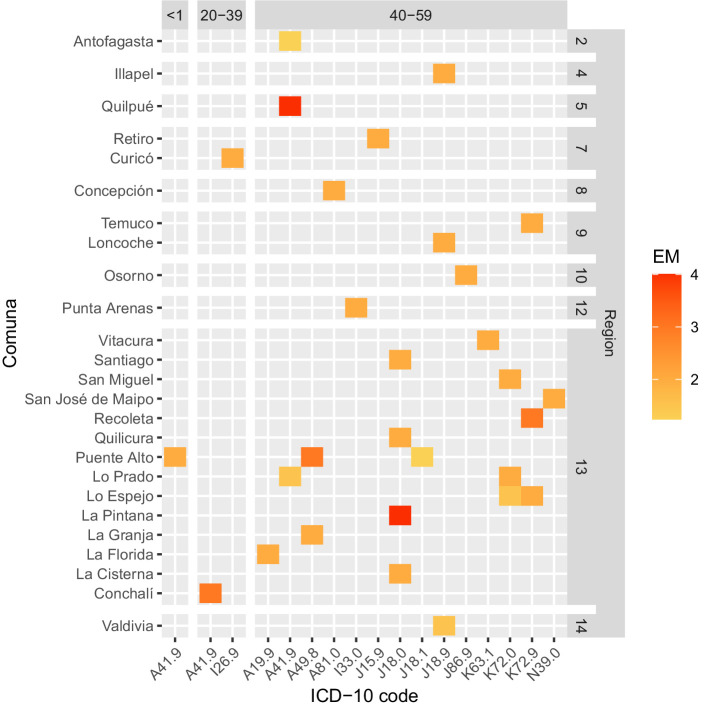
Heatmap depicting Comunas with excess of mortality (EM, ASMR/ASIR>1) by age group and ICD-10 code during 2023. Age groups included: under 1 year old, 20–39, and 40–59 years old. The color scale depicts low EM in yellow and high EM in red.

**Figure 5:**
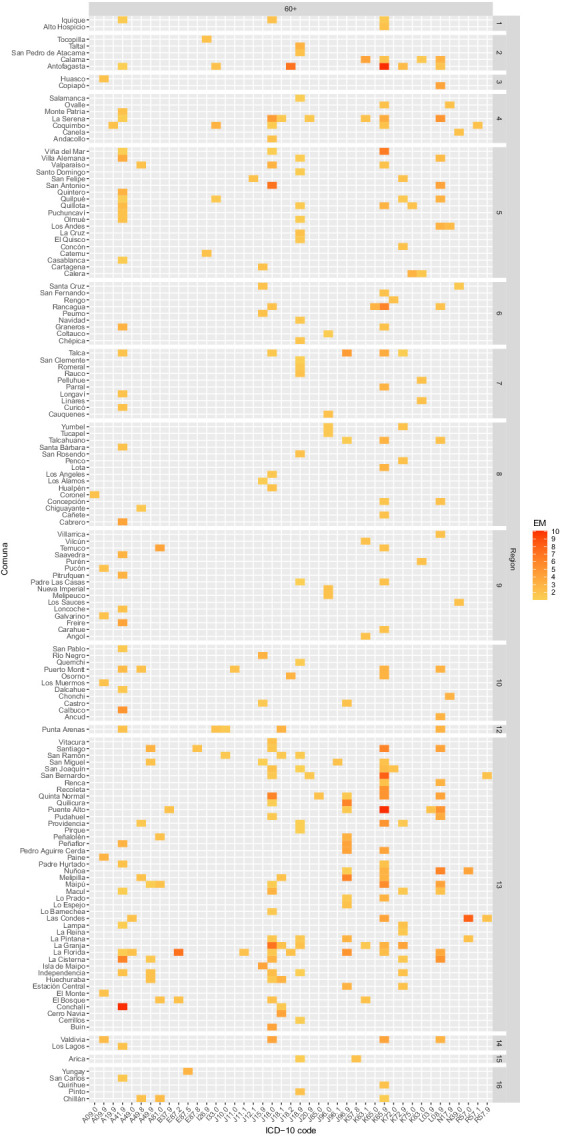
Heatmap depicting Comunas with excess mortality (EM, ASMR/ASIR>1) by age group and ICD-10 code during 2023. The age group included is 60 years and older. The color scale indicates low EM in yellow and high EM in red.

Sepsis-related EM was also calculated by individual ICD-10 code, allowing for setting a point of action for public health policies (Supplementary Table 13). Measured in this way, a heatmap was constructed to facilitate rapid interpretation of code-specific EM contributions and distributions by Region, Comuna, and age group (Figures 4, 5). The causes with the highest EM were found in the age group >60 years old, with peritonitis (K65.9) and septicemia (A41.9) leading the chart in Comunas Antofagasta, Conchalí, and Puente Alto (Figure 5 and Supplementary Table 13). Excluding the age group >60 years old, the causes with the highest EM were septicemia (A41.9) and bronchopneumonia (J18.0), leading the chart in Comunas Quilpué and La Pintana (Figure 4). Notably, no code-specific EM was detected in the Aysén Region.

### Sensitivity and uncertainty assessment

A global sensitivity analysis was performed using a Monte Carlo simulation, with population and sepsis-related figures for deaths and hospital discharges as inputs and expected deaths or discharges as outputs. Using the Spearman rank correlation method on the output from 10,000 Monte Carlo iterations and assessing it with a Spearman rank correlation, the sensitivity analysis revealed that the variation in the output was driven by uncertainty in population size [very high correlations at the region (*ρ* = 0.96–0.97) and Comuna (ρ = 0.83–0.84) levels] with age-standardized rates as a secondary driver [weak correlations at the region (ρ = 0.23–0.27) and Comuna (ρ = 0.47–0.48) levels] (Supplementary Figure 11).

Uncertainty was examined visually by highlighting the width of CIs at the Country (Supplementary Figure 7) and region (Supplementary Figure 8) levels, and by inspecting CIs in error bar charts at the Comuna level (Supplementary Figure 9). The width of CIs, calculated with a gamma/chi-squared approximation, was too narrow to appear visually. Then, uncertainty was evaluated region versus region and Comuna versus Comuna by appraising the variance of the difference between age-standardized rates using a Z-test. At the regional level, a greater proportion of significant combinatorial differences was observed for ASIR (89.2% significant differences) than for ASMR (68.3% significant differences). At the Comuna level, a greater proportion of significant combinatorial differences was observed for ASIR (77.3% significant differences) than for ASMR (26.7% significant differences). Results of these *Z*-tests were plotted in *p*-value matrix heatmaps to ease inspection by region (Supplementary Figure 12) and Comuna (Supplementary Figures 13, 14).

### Single code bias in EM

A significant limitation when using Chilean open-access datasets is the unavailability of MCOD. To assess bias in our SCOD approach to sepsis identification, we queried auxiliary open-access databases from OECD member countries. Given its cultural and socioeconomic similarities to the Chilean population and the quality, completeness, and comprehensiveness of its open-access data, Brazil’s MCOD dataframes were selected as external data to calculate a correction ratio (*λ*) to further adjust Chilean sepsis-related rates. For the MCODe approach, sepsis was identified as detailed in the methods section (Supplementary Figure 5). Correction ratio was calculated as the quotient between Brazilian sepsis-related MCODe ASMR and SCODe ASMR by sex and year. Computed as such, the relative bias in Chilean single-code ASMR ranged from 19.1 to 39.8% between 2015 and 2022, and from 22.5 to 25.8% during the last year analyzed, representing an average underestimation of 24.1% in 2023 (Supplementary Table 14 and Supplementary Figure 15).

Missing content in variables for the primary and secondary causes of hospitalization in Brazilian open-access discharge datasets was 41.2 and 97.2%, respectively (Supplementary Figure 4). Therefore, neither an MCODi- nor a single cause of discharge (SCODi)-based correction ratio λ could be calculated. Other sources of open-access SCODi were sought within OECD member countries. However, no instances were identified, rendering any attempt to calculate an MCODi/SCODi correction ratio λ unfruitful. Therefore, in the current open-access landscape, estimating single-code bias in EM is not computationally feasible.

### Randomness bias in missing data

Randomness bias was assessed only in discharge dataframes, following the dataframe structure, for which completeness was <99.95% (Supplementary Figure 2). Not available content in Country and region dataframes was observed to be perfectly matched across all incomplete variables. Therefore, no test could be performed. Undetermined content completeness was <99.95% only in the Comuna dataframe (99.26%). Chi-squared goodness-of-fit test revealed that differences across counts in age groups were not random (*p* < 0.001), favoring age groups 90 and older and 1–9 years old (Supplementary Table 15). Similarly, differences in counts across Comunas were not random (*p* < 0.001), favoring the use of code “99,999” (ignored) over “88,888” (foreign national), with both contributing equally to the total chi-squared statistic.

## Discussion

Public health systems in LMICs are underfunded and overburdened by high demand and are hence poorly resilient (20). Therefore, the efficient deployment of resources under scarcity is vital to withstand pressure while remaining effective. In this report we present a strategy that provides highly specific and actionable targets for public health interventions related to sepsis with the feature to diagnose specific and localized failures in the response of the health system to specific types of sepsis, while presenting a diagnosis about the burden of sepsis in Chile and presenting an argument supporting the use of granular data to guide the implementation of new healthcare policies from the deepest level of administration available.

In digital graphics, a raster is a digital image made up of a grid of pixels. The smaller the pixels, the greater the resolution. By analogy, in our results, a deeper level of analysis increased the resolution of sepsis-related rates. A clear contrast was evident side by side, with Comunas with a green tint appearing within others with a red tint, all constituting Regions otherwise dominated by red tints (Figures 3A,B). Moreover, higher resolution was achieved by disaggregating the data by age group (Supplementary Figure 10), revealing that some Comunas remained unaffected by sepsis. Thus, even finer resolutions may be achieved by using district- or neighborhood-level data, an approach that could uncover underlying determinants extending beyond the domain of health (e.g., low rural connectivity, transportation costs, distribution of health centers, etc.). Therefore, we advocate for public health policies to be conceived and shaped using data of optimal resolution, balancing small-area data with estimation stability. Additionally, our integrative approach highlights that measuring mortality or incidence alone offers a limited view of the subnational burden and overall status of sepsis, or –by extension– of any other disease. Measurement of sepsis-related EM provides a starting point for public health policies by signaling locations where mortality exceeds incidence, or, in lay terms, places where sepsis is not being contained by the health system, all with a Comuna-level of resolution. The latter, coupled with EM by cause of death, offers an unprecedented method and opportunity to tackle sepsis efficiently and effectively.

Quality control in our analyses considered assessing NA and undetermined content, sensitivity, and uncertainty. Quality of open access data retrieved from Chilean DEIS was consistent with prior reports underscoring Chile’s official civil registration and vital statistics (9, 10), with an overall completeness of >96.8%, an inverse correlation between population size and rate sensitivity (a fact further supporting our argument about the use of high-resolution data to shape public health policies), and presence of narrow CIs for all rates calculated. Owing to the quality of the data, the assessment of uncertainty enables the translation of results into plausible interpretations. First, the higher proportion of significant pairwise comparisons for ASIR compared to ASMR both at the region (Supplementary Figure 12) and Comuna levels (Supplementary Figures 13, 14) is consistent with the inherent circumstance that a hospital discharge with an «alive» outcome is much more likely than with a «dead» outcome, increasing the base of events for ASIR calculations, leading to a smaller sampling variance than for ASMR calculations. Second, because the proportion of significant pairwise comparisons for ASIR was ~89% and for ASMR was ~68%, it may be inferred that the risk of developing sepsis varies considerably across Regions, whereas the risk of dying from sepsis is more homogeneous across Regions. Strikingly, as evidenced from the proportion of significant pairwise comparisons for ASIR (~77%) and ASMR (~27%) at the Comuna level, the risk of developing sepsis remains highly variable from Comuna to Comuna, but the risk of dying from sepsis is dramatically more coherent across them. This accounts for the homogeneity of healthcare responses within the country, not just underlining how uniform the standard of treatment for sepsis is across Comunas, but also signaling that current (by 2023) success lies in the competence of healthcare rather than in prevention efforts. Therefore, a combined approach by the central government involving (1) strategic planning to allocate major resources and target high-risk Regions for massive intervention, and (2) operational planning to evaluate local programs and compare nearby healthcare centers, should be the fundamental *modus operandi* to achieve progress in reducing the burden of sepsis in Chile, and possibly the rest of Latin America (20, 21).

Presentation of EM in heatmaps (Figures 4, 5) is a distinctive feature of our analysis. While, at first, the simplest form of interpretation is its ability to display sepsis-related underlying causes of death in a geo-*aetas* fashion, more complex interpretations could be drawn about healthcare singularities (e.g., incidental shortage of staff or beds, or an outbreak of antibiotic-resistant strains) if EM is scattered across Comunas and about healthcare problems that are more systematic (i.e., from failure in Regional resources or policies) if EM is found constant across Comunas. Moreover, filtering interventions by ASMR/ASIR ratio and targeting ICD-10 codes with EM theoretically enables the maximization of the number of lives saved per intervention unit, a crucial feature for enhancing resilience under resource scarcity, while providing precise information about the intervention to implement. Thus, for pneumonia-related sepsis, a Regional campaign to improve vaccination or to implement rapid diagnostic X-ray protocols for elderly patients appears to be a sound intervention, centered on prevention rather than treatment, tackling sepsis-related ASIR directly and ASMR indirectly. Similarly, interventions to control infection-associated causes may include the implementation or upgrade of antibiotic resistance surveillance, the enhancement of source control, the use of empiric treatment protocols, the operationalization of targeted clinical audits to evidence potential delays in antibiotic treatment or missed early warning signs, and the redirection of quality standards based on code-specific EM magnitude. Likewise, intervention bundles, including the relocation of infectious disease and critical care specialists, increasing ICU bed capacity, bedside equipment, and staffing, and/or improving laboratory capacity for rapid culture identification, may be proposed, Comuna- and Region-wise. Furthermore, presence of codes with an undetermined or unspecified component [e.g., Peritonitis, unspecified (K65.9), septicemia, unspecified (A41.9), Bronchopneumonia, unspecified (J18.0), etc.] in Figures 4, 5, may indirectly signal a preponderance of fulminant events, an incapacity to identify the causative agent on time, or a lack of specificity by healthcare professionals in charge of filling the death certificate, opening the door for code misuse audits.

There are few robust health-related databases globally to assess the burden and impact of sepsis. Fleischmann-Struzek and Rudd (22) Latin America offers an incalculable opportunity to implement data-driven health policies because, among its countries, vital registration data is maintained with open access by government agencies with a high level of detail and an overall quality ranging from medium to high (9). However, analyses using datasets from Latin American OECD member countries, such as Brazil, Colombia, and Mexico, reveal that completeness of death records (NA content <5%) is much higher than that of hospital discharge records (NA content up to 98.2%) (Supplementary Figure 4). Unlike Chilean death and hospital discharge records (10), these incomplete datasets cannot be used as input to our strategy, severely compromising its international comparability and scalability when relying on open-access datasets. Therefore, the present study emphasizes the need to improve vital registration in the region and calls for equitable open-access data to promote research.

A plethora of articles have reported the burden of sepsis in terms of either mortality or incidence. Some have described such a burden in terms of estimations, of which the article by Rudd et al. (2) stands out for its comprehensiveness (reporting sepsis-related incidence and mortality globally, by region, and by country) and for its pioneering strategy to define sepsis-related cases in terms of ICD codes. Although trends in the latter study are not directly comparable to ours because of a difference in timeframes (1990–2017 vs. 2015–2023), estimates for 2017 provide an interesting point of comparison. By means of complex modelling algorithms, using official records from Austria, Brazil, Canada, Chile, Georgia, Italy, Mexico, New Zealand, Philippines, and the United States, as input for modelling ASIR, and official records from Brazil, Taiwan, the United States, and Mexico, as input for modelling ASMR, the estimated ASIR and ASMR for Chile in 2017 were 260.8 and 52.6 per 100,000 population, respectively. Compared to our calculations based on official records, an 80% and an 11.4% underestimation was found for ASIR and ASMR (or a 30.1% underestimation for ASMR, considering correction by external data (Supplementary Figure 15). Thus, these imprecisions illustrate the unsuitability of estimates to guide local (i.e., county-by-county, state-by-state, and country-by-country) public health policies, particularly when official civil registration and vital statistics systems for death registration and medically certified causes of death are of high quality. Paradoxically, countries with high-quality data offer no easy access to raw data, making academic analysis challenging. Accordingly, very few studies (from LMICs, incidentally) have analyzed the subnational sepsis incidence and mortality from official records. Although with limited coverage for hospital discharge (64.1% of all tertiary hospitals and 22.7% of all secondary hospitals in mainland China) and death records (24.3% of coverage for mainland China), a high proportion of exclusions due to missing data 11.2% of hospitals due to missing unique identifier followed by an additional ~13.2% of admissions due to exclusion criteria), and use of a prior version of ICD-10, article by Weng et al. (23) highlights geographical disparities and shows that the gain in use of MCOD for the identification of sepsis among hospital admissions was ~19% (close to our calculation of 24.1% underestimation for SCODe, Supplementary Table 14 and Supplementary Figure 15), that deaths by sepsis accounted for 13.1% of all deaths in the study period (i.e., 2017–2019), a number close to calculations in our study (9% for 2015 to 13.7% for 2023), that a higher proportion of sepsis incidence affects the elderly (unlike with our study, Supplementary Figure 6), that sepsis ASIR rose from ~419/~269 (males/females) to ~519/~343 per 100,000 population, (in contrast to our calculations of ~1,366/~1,438 and ~1,415/~1,435 per 100,000 population, respectively) and that sepsis ASMR rose from ~24/~12 (males/females) to ~27/~13 per 100,000 population (in contrast to our calculations of ~54/~65 and ~76/~80 per 100,000 population, respectively). Another study highlights progress in tackling sepsis in Latin America by using official records of hospital discharge and death-related sepsis from the Brazilian predecessor of DATASUS. Although from 2011, covering 80% of national hospital admissions, detailing a very narrow ICD code list used to identify sepsis, not clarifying data exclusion, and focusing only on pediatrics, the article by Mangia et al. (24) showed trends of reduction of hospital admissions and hospital sepsis mortality between 1992 and 2006.

## Limitations

A significant limitation of our study was the unavailability of MCOD. Assessment of bias from the use of SCODe found an underestimation of sepsis-related deaths of 19.1–39.8% (between males and females), with an average of 24.1% in 2023 (Supplementary Figure 15). While this magnitude might seem high, the underestimation of SCODi is likely similar to that observed when open-access MCODi datasets are available. Therefore, it is plausible to expect EM calculations to balance out (to an immeasurable extent at present) without a significant change in the output. Two scenarios branch from this ineluctable circumstance: either the present strategy is refrained from publication until external open access data is available in adequate form, contributing to the “[lack of] data available from the Americas” (13) by remaining static, or—based on the precautionary principle, which states that “with a serious possibility of danger [i.e., higher risk of death by sepsis], communities are advised to alter their behavior without the necessity of a high degree of scientific proof that would normally be required to validate an intervention” (25)—acknowledge this gap in certainty, apply a cautionary disclaimer for interpretation, and address access to MCODi diplomatically instead of academically. We feel compelled to follow the latter.

Another limitation is the inability to address potential confounders for temporal trends (e.g., changes in coding practices, healthcare access, or diagnostic capabilities over the study period) because, to the best of our knowledge, there is no documentation of such changes. Although a statement announcing the rejection of Sepsis-3 (1) was found on the website of the Chilean Society of Intensive Care (*Sociedad Chilena de Medicina Intensiva*, SOCHIMI) (26), no single official document reflecting a change in sepsis-related coding practices was found.

Further limitations include possible inaccuracies in code use, the lack of a validation set, and the absence of morbidity data. First, Chile adheres to PAHO’s CVRS programs (27, 28) and offers continuous training to clinicians through different courses, updated standards, recommendations, and guidelines, via the National Reference Center for the Family of International Classifications (*Centro Nacional de Referencia de la Familia de las Clasificaciones Internacionales*), based in the Undersecretariat of Public Health (*Subsecretaría de Salud Pública*) and implemented by the DEIS. Second, access to clinical records (for validation) is restricted by laws N° 19.628 and N° 21.719 (29, 30), which introduce reforms to align Chile’s data protection framework with international standards [e.g., the European Union’s General Data Protection Regulation (31)]. Although negotiations to access such data have been initiated on our behalf with the relevant authorities (in 2023), this ongoing process carries no guarantee of success. The latter extends to morbidity data as well.

Although the randomness of undetermined content, favoring age groups 90 and older and 1–9 years old (Supplementary Table 15), found in the Comuna dataframe of hospital discharges may constitute a limitation to our interpretations, the extent of such non-randomness bias is virtually negligible, representing 0.7% of the dataframe.

## Conclusion

In conclusion, we present a data science methodology to identify subnational areas where sepsis-associated mortality exceeds its incidence, which may be replicated using data from any country with reliable hospital discharge and death records. The output of this methodology enables prioritization of government resources by the magnitude of the excess, allowing for a more efficient approach to tackling sepsis, especially in LMICs.

## Data Availability

The datasets analyzed in this study were found and may be accessible in the official websites of the Departamento de Estadísticas e Información de Salud, (DEIS), available at: https://deis.minsal.cl, and the Instituto Nacional de Estadísticas (INE), available at: https://www.ine.gob.cl/estadisticas/sociales/demografia-y-vitales/proyecciones-de-poblacion. Processed datasets are included as supplementary material. Further inquiries may be directed to the corresponding author.

## Supplementary material

SUPPLEMENTARY FIGURE 1 Algorithm of inclusion/exclusion for Chilean death datasets used in this study.

SUPPLEMENTARY FIGURE 2 Algorithm of inclusion/exclusion for Chilean hospital discharge datasets used in this study.

SUPPLEMENTARY FIGURE 3 Algorithm of inclusion/exclusion for Brazilian death datasets used in this study.

SUPPLEMENTARY FIGURE 4 Algorithm of inclusion/exclusion for Brazilian hospital discharge datasets used in this study.

SUPPLEMENTARY FIGURE 5 Definition of sepsis based on single (SCOD) or multiple cause of death/discharge (MCOD) approaches **(A)**. Illustration of external data (Brazil death datasets) structure and application of SCOD/MCOD **(B)**.

SUPPLEMENTARY FIGURE 6 Ranking and progression of sepsis-related age-standardized incidence rate (ASIR) (**A**, males; **B**, females) and age-standardized mortality rate (ASMR) (**C**, males; **D**, females), along with 22 ICD chapters by age group in Chile from 2015 to 2023.

SUPPLEMENTARY FIGURE 7 Progression of the sepsis-related age-standardized incidence rate (ASIR) **(A)** and age-standardized mortality rate (ASMR) **(B)** by sex in Chile from 2015 to 2023.

SUPPLEMENTARY FIGURE 8 Progression of sepsis-related age-standardized incidence rate (ASIR) **(A)** and age-standardized mortality rate (ASMR) **(B)** by region in Chile from 2015 to 2023.

SUPPLEMENTARY FIGURE 9 Progression of sepsis-related age-standardized incidence rate (ASIR) **(A)** and age-standardized mortality rate (ASMR) **(B)** by region and Comuna in Chile from 2015 to 2023. See Supplementary Table 4 for the key for Comuna.

SUPPLEMENTARY FIGURE 10 Geographic distribution of sepsis-related age-standardized incidence rate (ASIR), age-standardized mortality rate (ASMR), and excess of mortality (EM), during 2023 by age.

SUPPLEMENTARY FIGURE 11 Tornado plot for Spearman rank correlation of the output from Monte Carlo simulations to assess the sensitivity of age-standardized incidence (ASIR) and mortality (ASMR) rates by region and Comuna.

SUPPLEMENTARY FIGURE 12 *P*-value matrix heatmaps of Z-tests to assess the variance of the difference between age-standardized incidence (ASIR) and mortality (ASMR) rates by Region.

SUPPLEMENTARY FIGURE 13 *P*-value matrix heatmaps of Z-tests to assess the variance of the difference between age-standardized incidence rates (ASIR) by Comuna.

SUPPLEMENTARY FIGURE 14 *P*-value matrix heatmaps of Z-tests to assess the variance of the difference between age-standardized mortality rates (ASMR) by Comuna.

SUPPLEMENTARY FIGURE 15 Comparison of sepsis-related age-standardized mortality rate (ASMR) progression in Chile from 2015 to 2023 by approach to define sepsis. The current approach is based on the single-cause-of-death (SCODe) definition. The corrected approach is based on recalculating SCODe using a correction factor *λ* derived from representative external data.

SUPPLEMENTARY TABLE 1 Type of coding for age in the root dataframes of death, discharge, and population.

SUPPLEMENTARY TABLE 2 List of ICD-10 codes used to identify sepsis in this study.

SUPPLEMENTARY TABLE 3 Processed the dataframe of the Chilean population by sex and age group. Standard population corresponds to the 2025 projection.

SUPPLEMENTARY TABLE 4 Processed a dataframe of the Chilean population by Region, Comuna, sex, and age group. Standard population corresponds to the 2025 projection.

SUPPLEMENTARY TABLE 5 Processed the dataframe of the Brazilian population by sex and age group. Standard population corresponds to the 2024 projection.

SUPPLEMENTARY TABLE 6 Processed dataframe of Chilean crude and age-standardized incidence rate (ASIR) by year and sex.

SUPPLEMENTARY TABLE 7 Processed dataframe of Chilean crude and age-standardized incidence rate (ASIR) by Region and year.

SUPPLEMENTARY TABLE 8 Processed dataframe of Chilean crude and age-standardized incidence rate (ASIR) by Comuna and year.

SUPPLEMENTARY TABLE 9 Processed dataframe of Chilean crude and age-standardized mortality rate (ASMR) by year and sex.

SUPPLEMENTARY TABLE 10 Processed dataframe of Chilean crude and age-standardized mortality rate (ASMR) by Region and year.

SUPPLEMENTARY TABLE 11 Processed dataframe of Chilean crude and age-standardized mortality rate (ASMR) by Comuna and year.

SUPPLEMENTARY TABLE 12 Processed dataframe of the quotient between Chilean age-standardized mortality rate (ASMR) and age-standardized incidence rate (ASIR) by year, Comuna, and age group.

SUPPLEMENTARY TABLE 13 Processed dataframe of the quotient between Chilean age-standardized mortality rate (ASMR) and age-standardized incidence rate (ASIR) by age group, Region, Comuna, ICD-10 code, and sepsis definition.

SUPPLEMENTARY TABLE 14 Processed dataframe of the quotient between Chilean age-standardized mortality rate (ASMR) and Brazilian ASMR (correction factor λ), with which a corrected Chilean ASMR was calculated. Bias from single cause of death (SCODe) use is expressed in absolute and relative terms.

SUPPLEMENTARY TABLE 15 Output of chi-square goodness-of-fit tests performed on undetermined cases in the variables age group and Comuna before exclusion from the Comuna hospital discharge dataframe (Supplementary Figure 2).
