# Supplementary figures and images for: A data science-led strategy to assess the subnational burden of sepsis using official records: a longitudinal description and cross-sectional demonstration in Chile
Source: Front Med (Lausanne). 2026 Jan 12;12:1671206. doi: 10.3389/fmed.2025.1671206 (PMC12832715; doi:10.3389/fmed.2025.1671206)

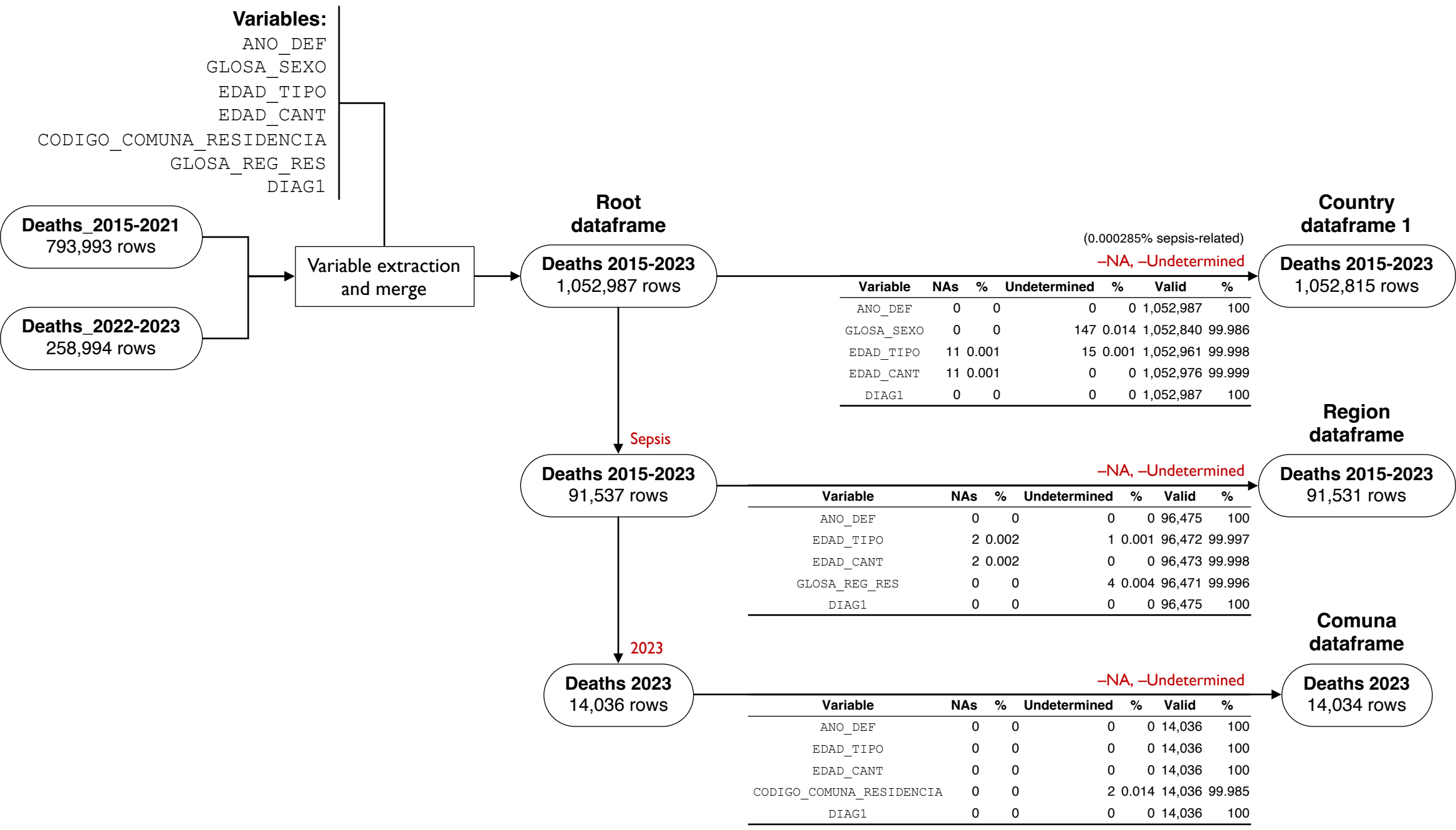

Supplement: SUPPLEMENTARY FIGURE 1 — Algorithm of inclusion/exclusion for Chilean death datasets used in this study. [file Supplementary_Image_1.pdf]

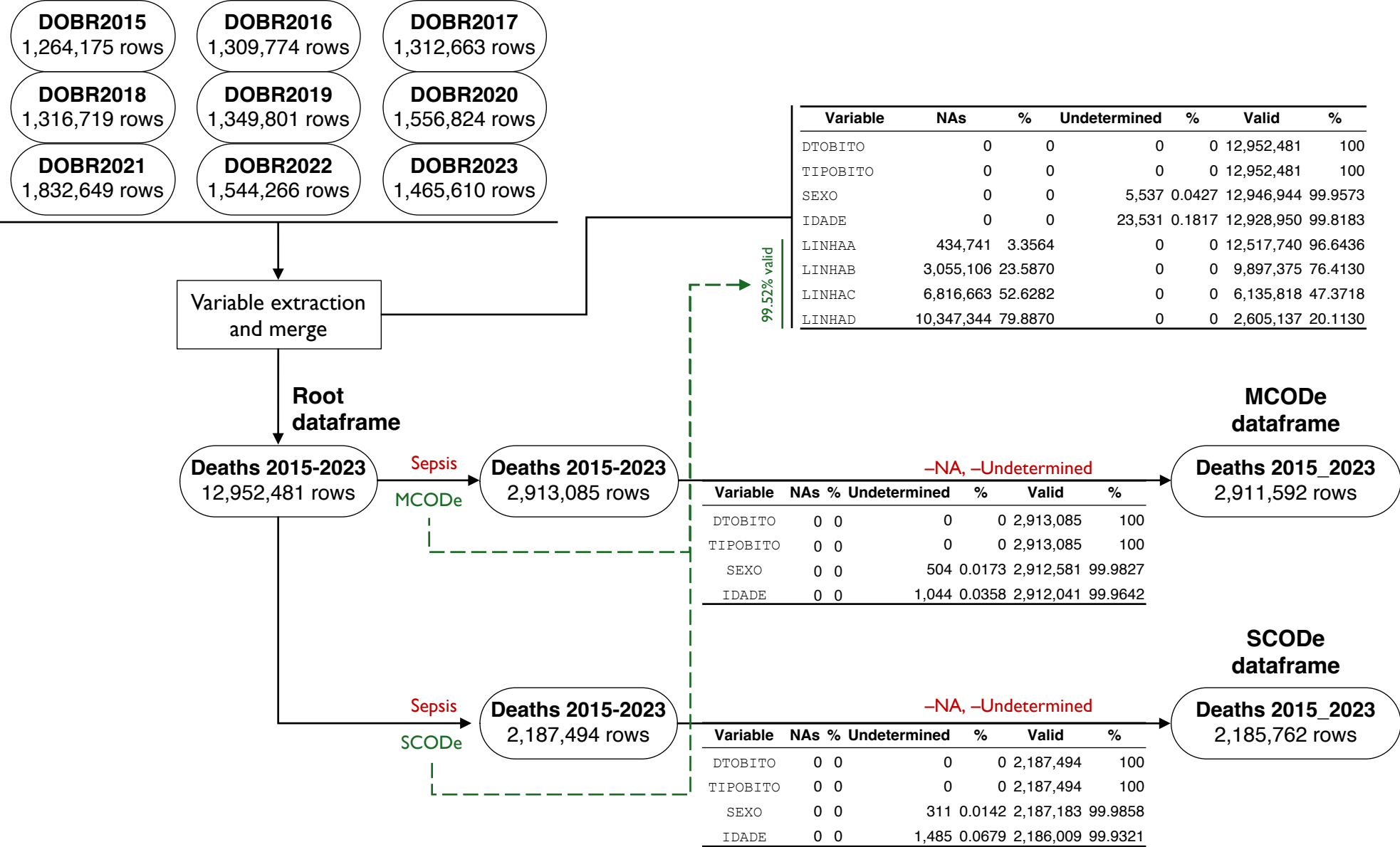

Supplement: SUPPLEMENTARY FIGURE 3 — Algorithm of inclusion/exclusion for Brazilian death datasets used in this study. [file Supplementary_Image_3.pdf]

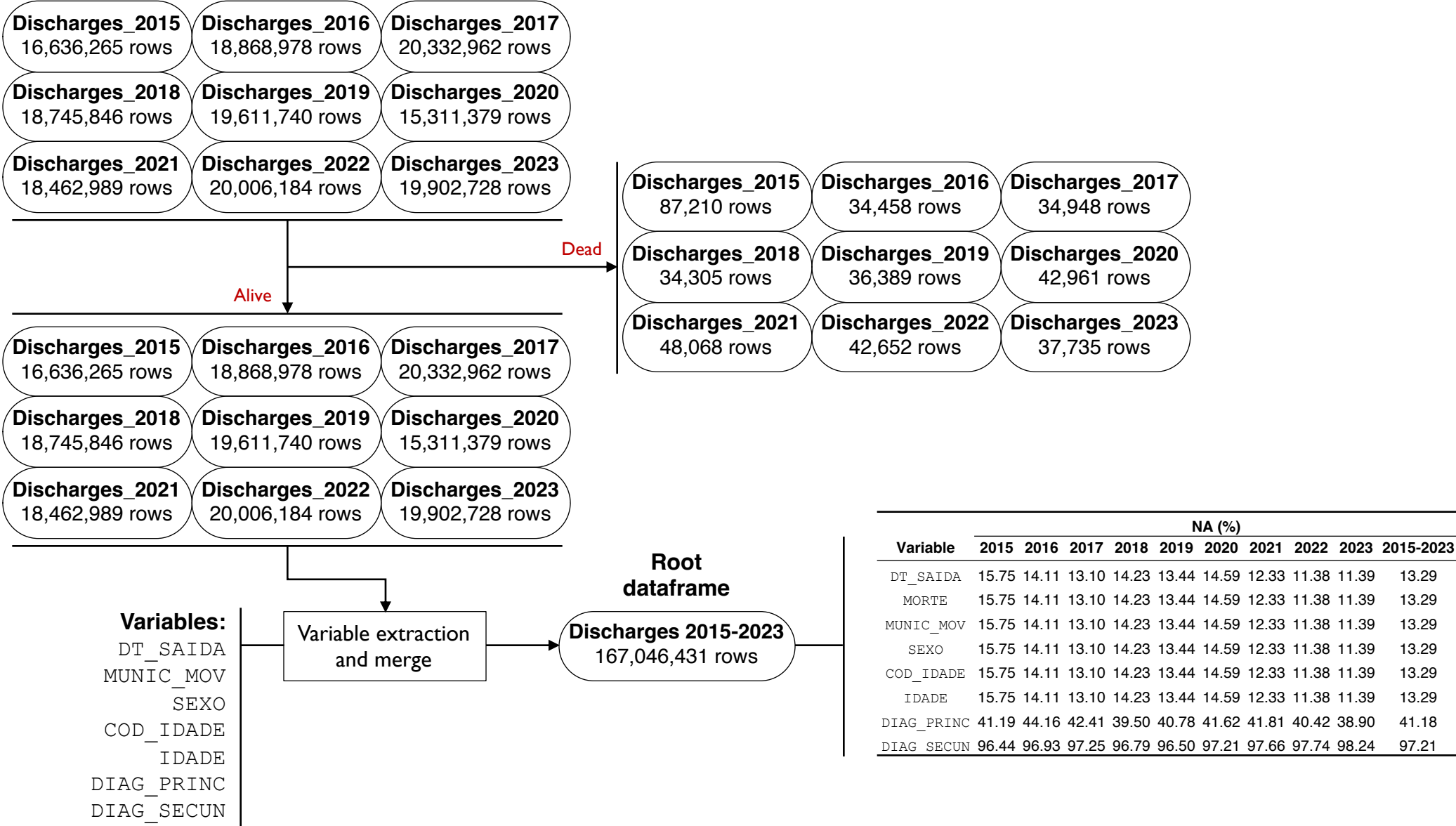

Supplement: SUPPLEMENTARY FIGURE 4 — Algorithm of inclusion/exclusion for Brazilian hospital discharge datasets used in this study. [file Supplementary_Image_4.pdf]

**A)**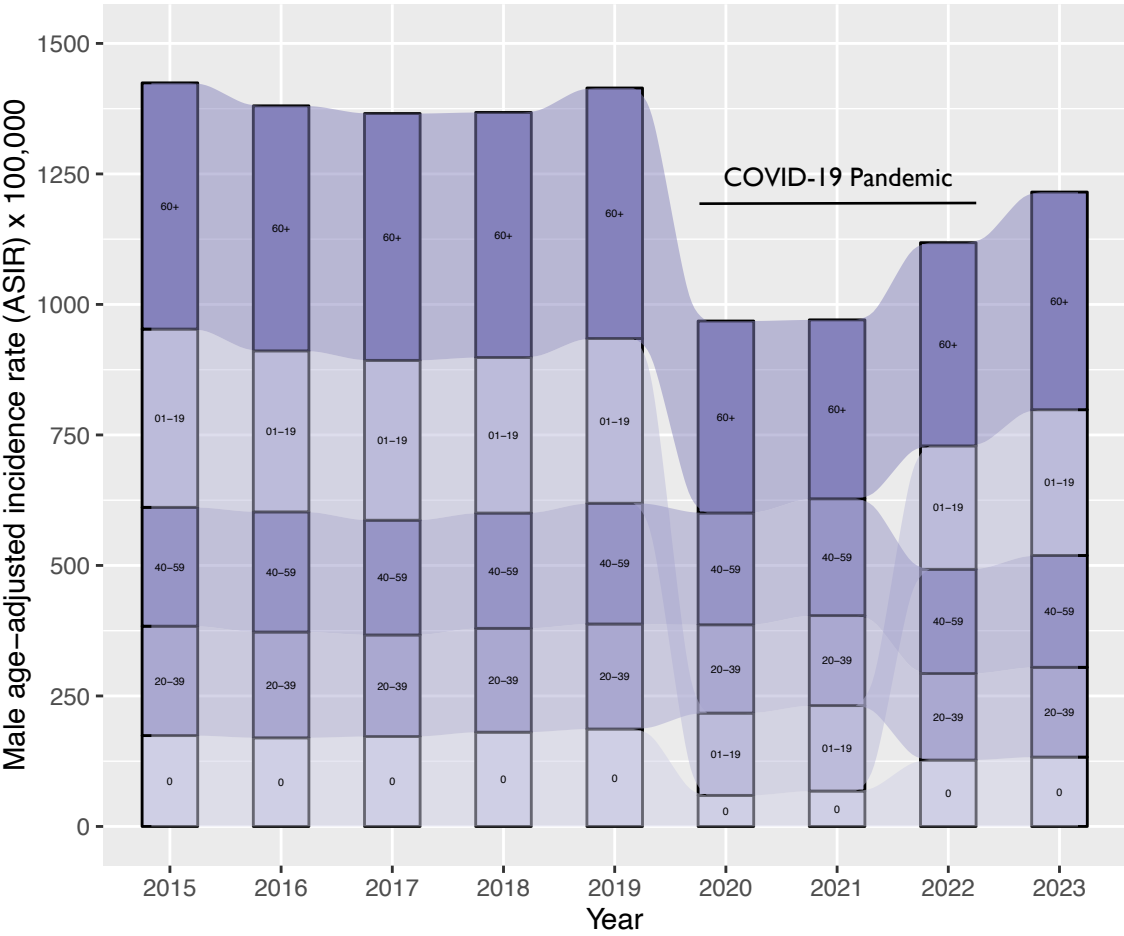**B)**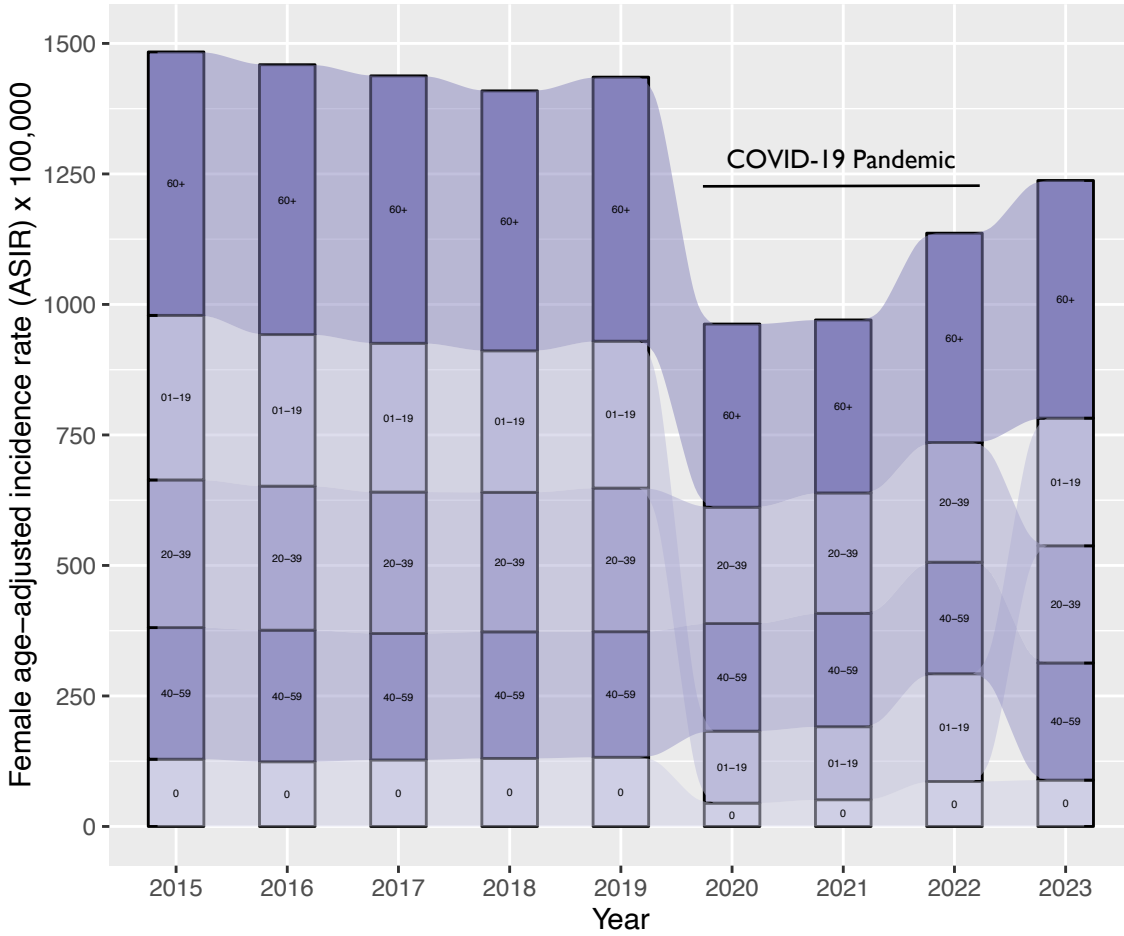**C)**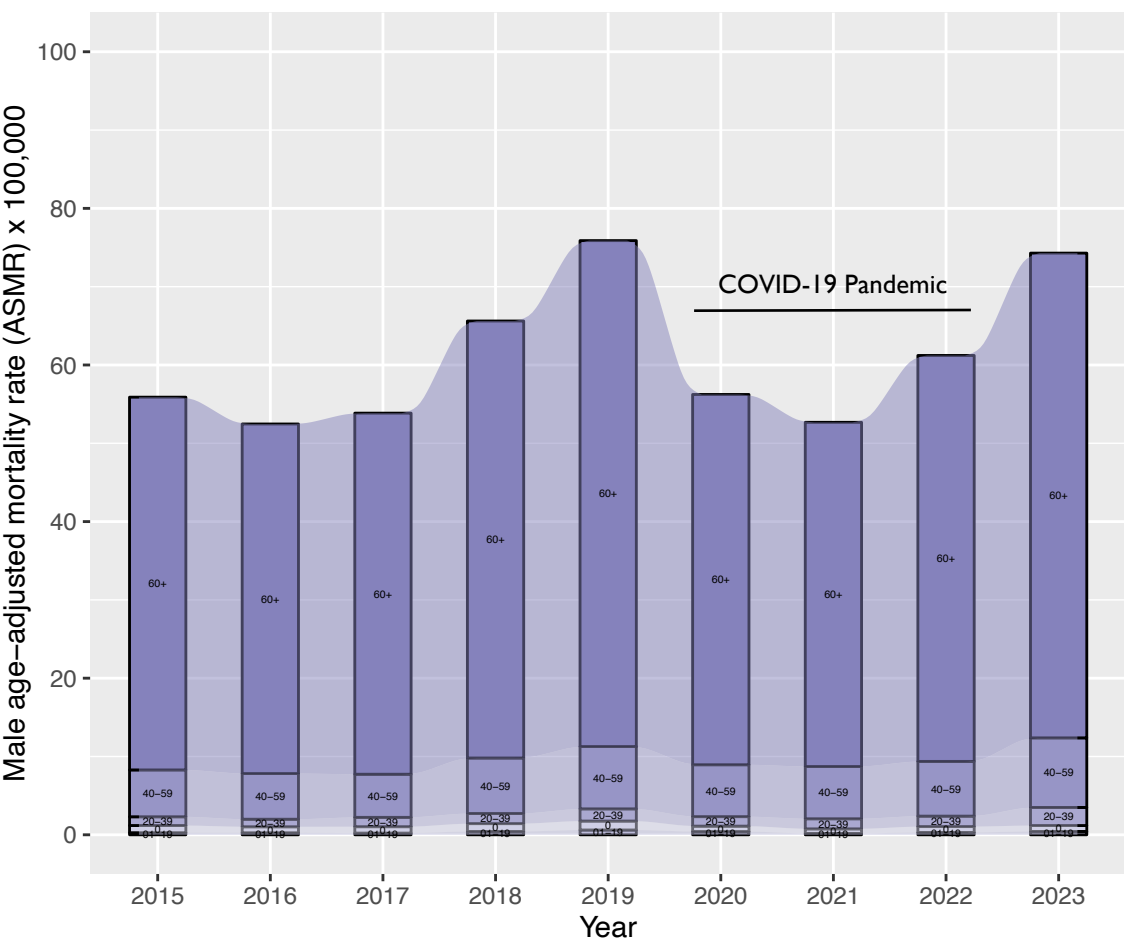**D)**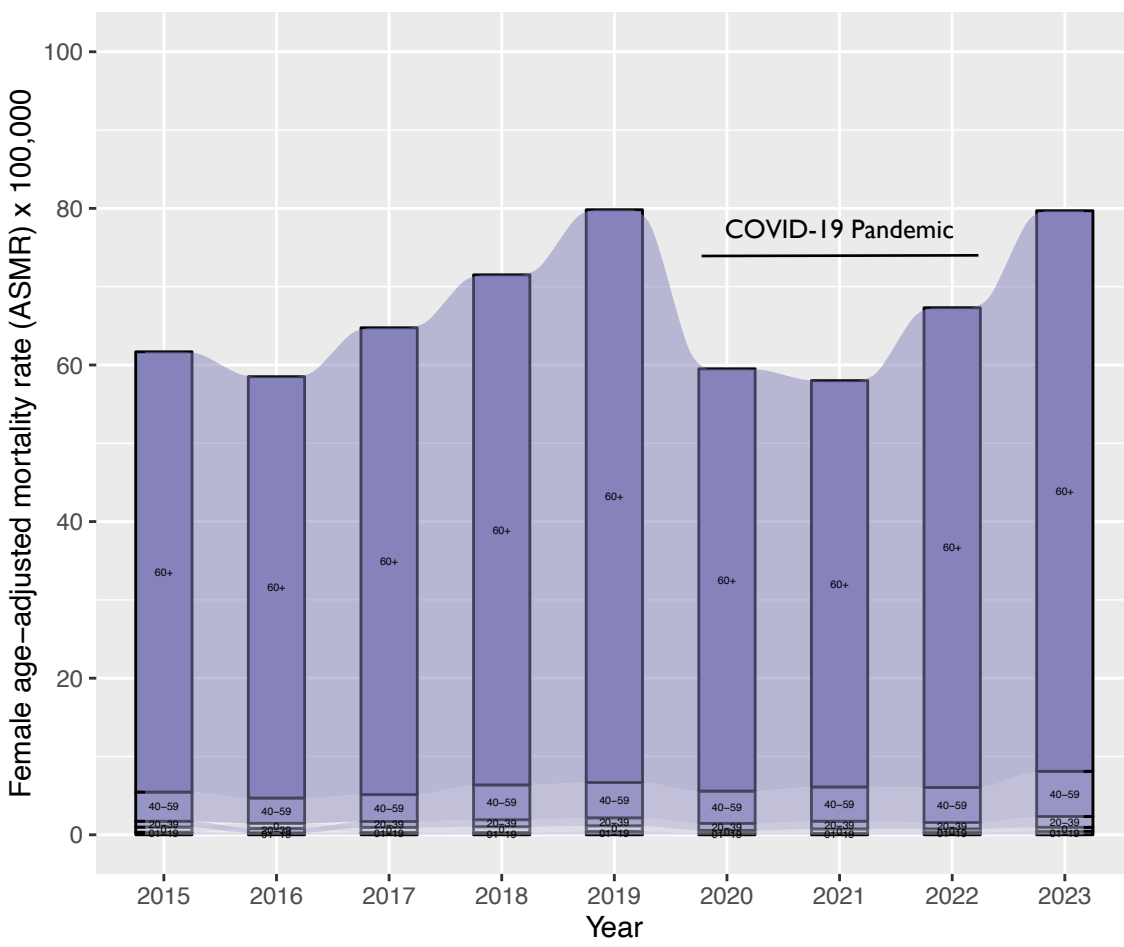

Supplement: SUPPLEMENTARY FIGURE 6 — Ranking and progression of sepsis-related age-standardized incidence rate (ASIR) (A, males; B, females) and age-standardized mortality rate (ASMR) (C, males; D, females), along with 22 ICD chapters by age group in Chile from 2015 to 2023. [file Supplementary_Image_6.pdf]

**A)**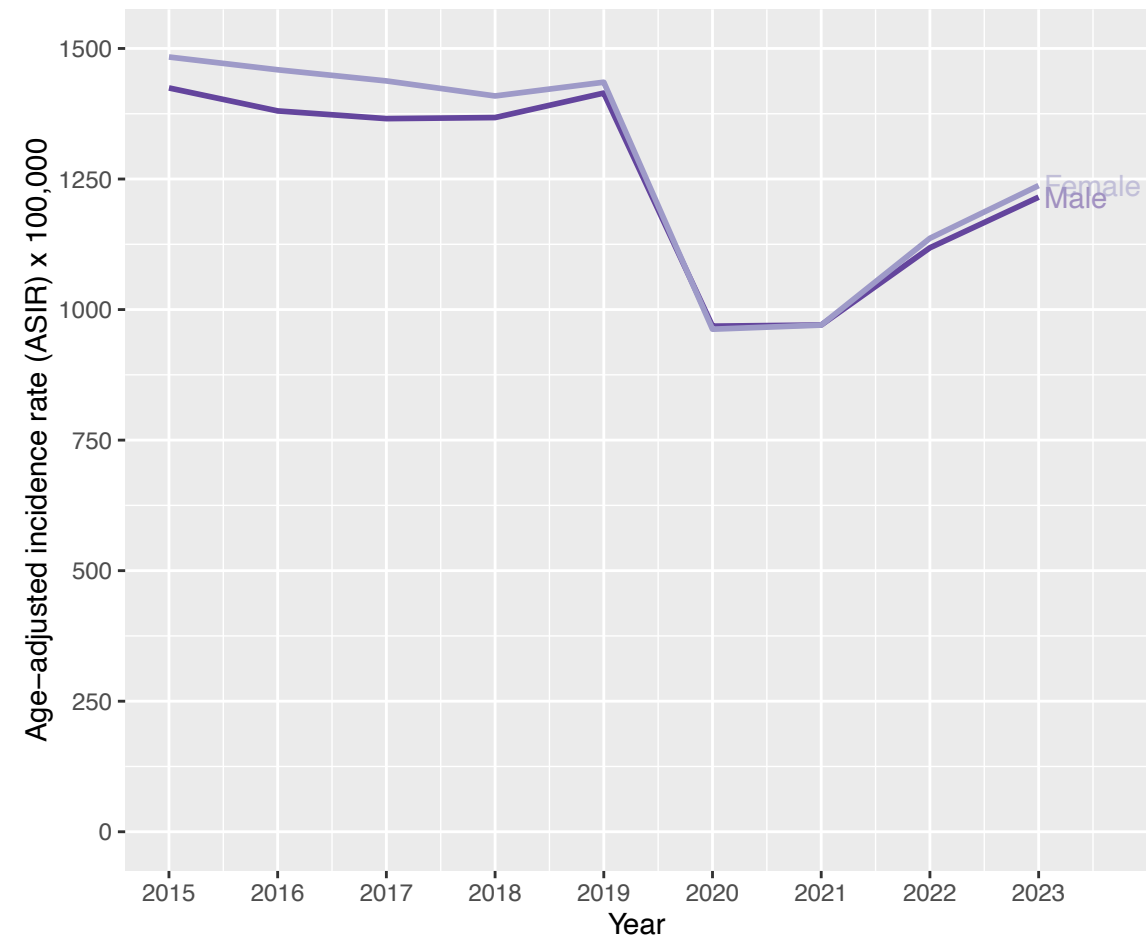**B)**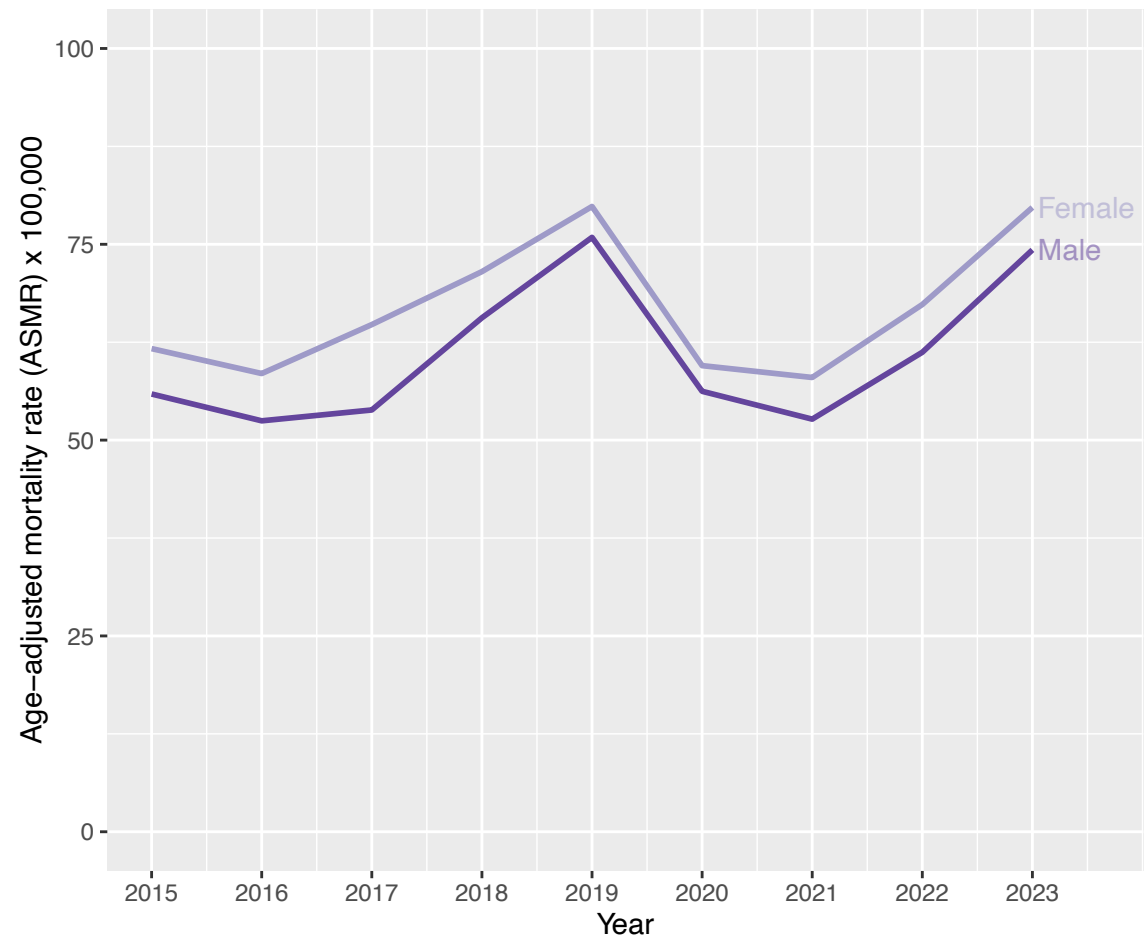

Supplement: SUPPLEMENTARY FIGURE 7 — Progression of the sepsis-related age-standardized incidence rate (ASIR) (A) and age-standardized mortality rate (ASMR) (B) by sex in Chile from 2015 to 2023. [file Supplementary_Image_7.pdf]

**A)**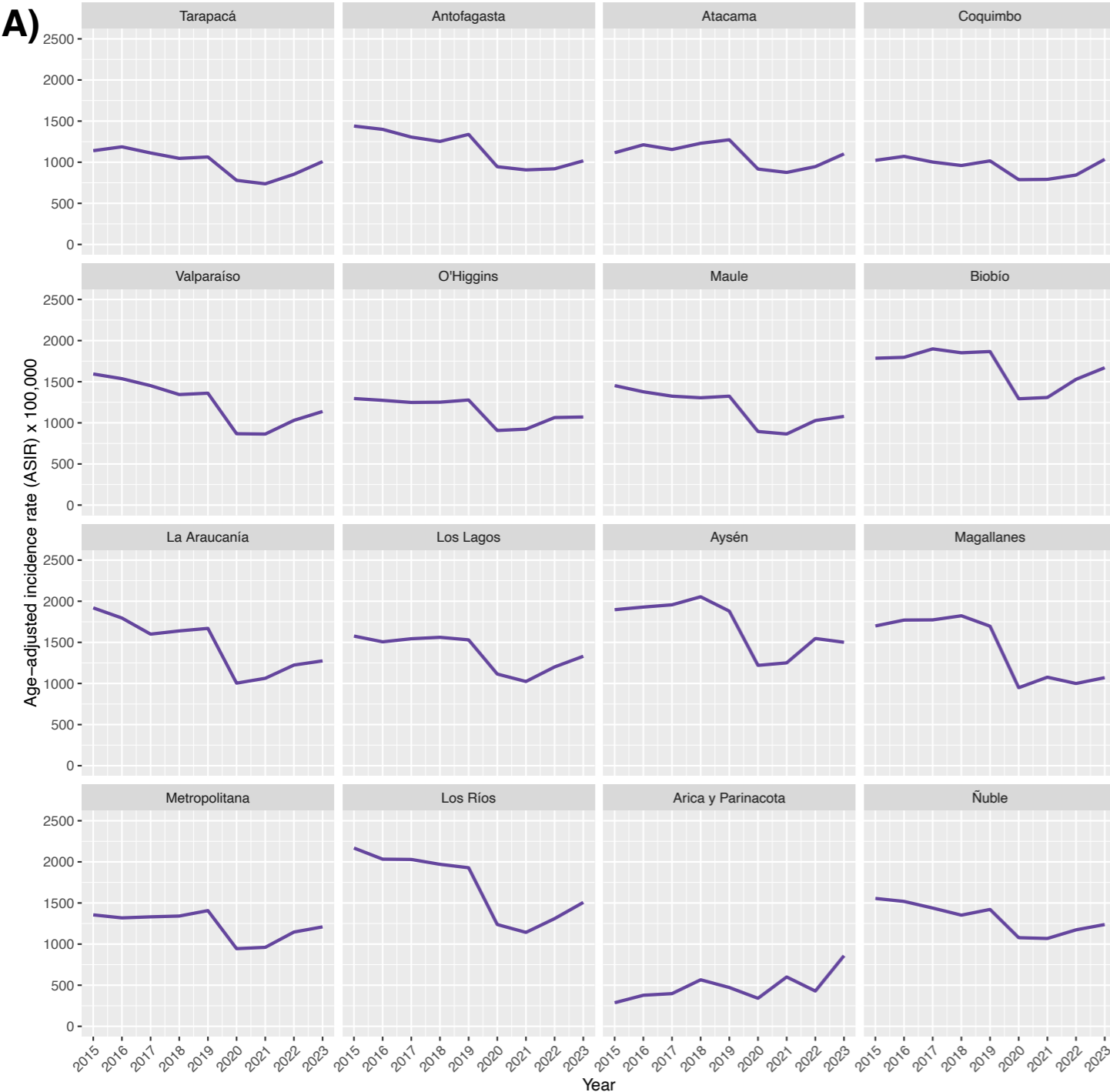**B)**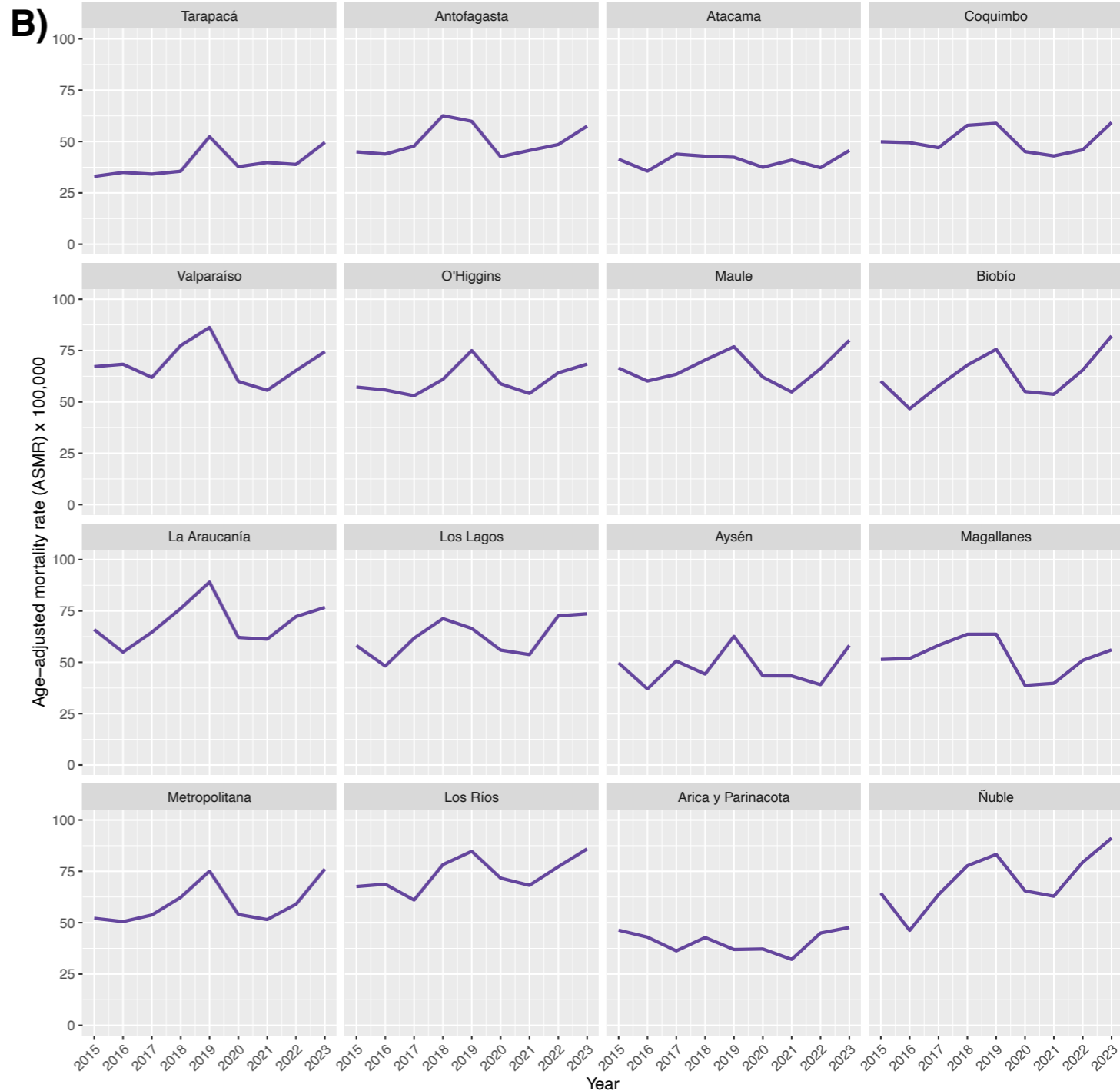

Supplement: SUPPLEMENTARY FIGURE 8 — Progression of sepsis-related age-standardized incidence rate (ASIR) (A) and age-standardized mortality rate (ASMR) (B) by region in Chile from 2015 to 2023. [file Supplementary_Image_8.pdf]

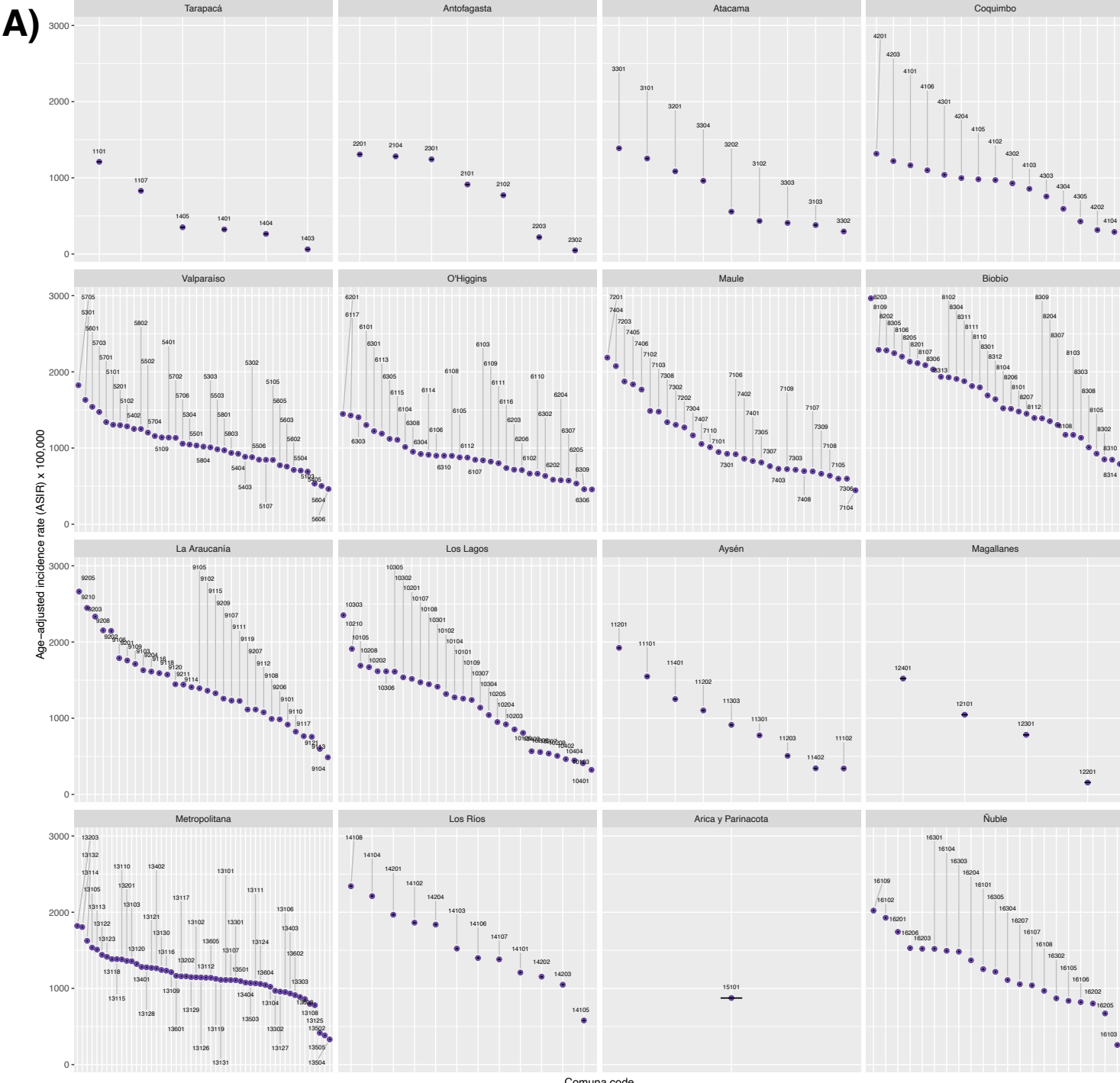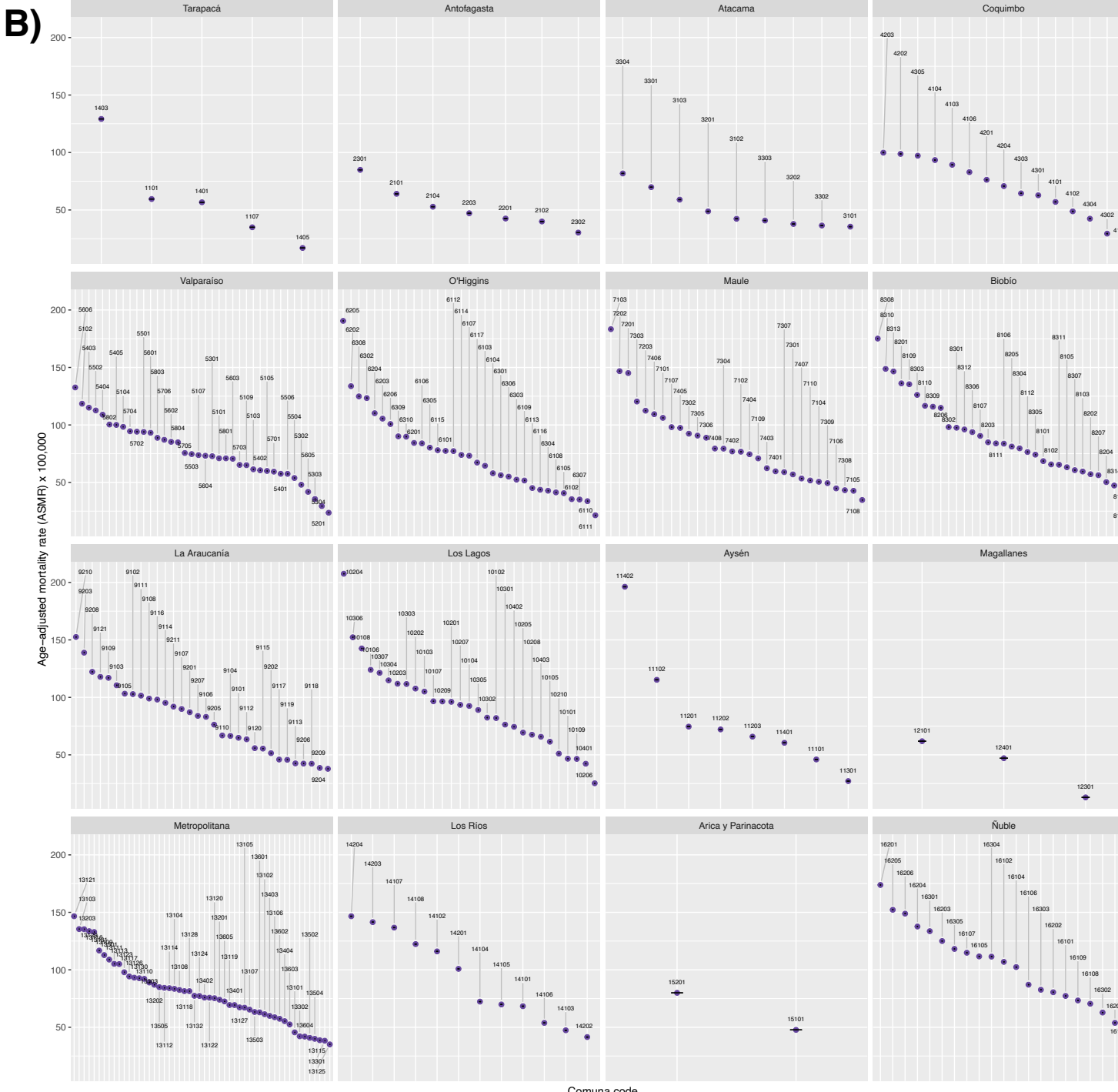

Supplement: SUPPLEMENTARY FIGURE 9 — Progression of sepsis-related age-standardized incidence rate (ASIR) (A) and age-standardized mortality rate (ASMR) (B) by region and Comuna in Chile from 2015 to 2023. See Supplementary Table 4 for the key for Comuna. [file Supplementary_Image_9.pdf]

## Incidence

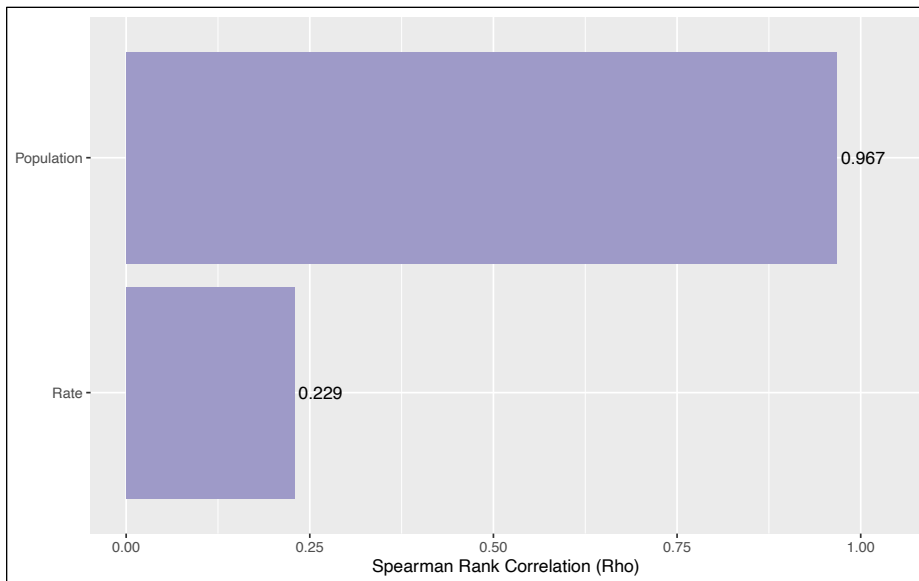

## Mortality

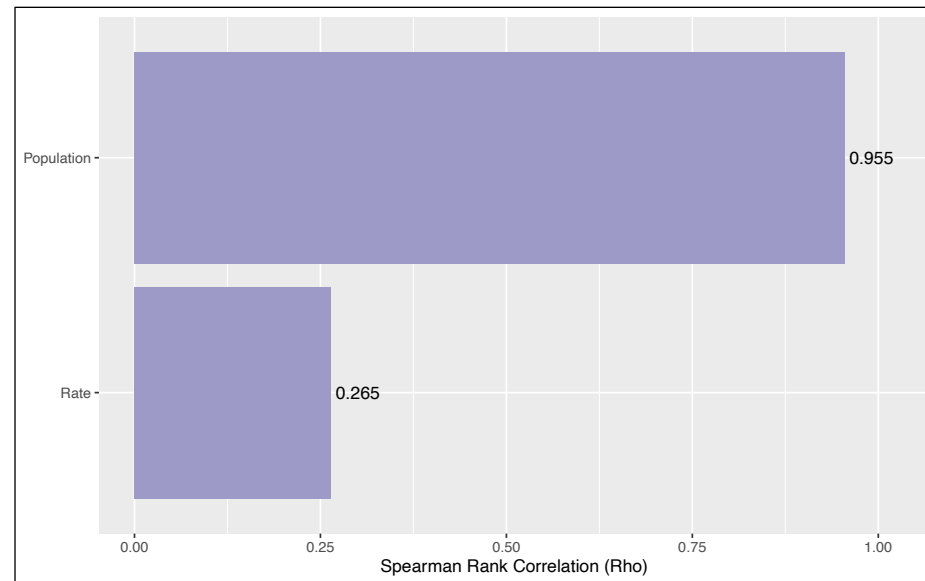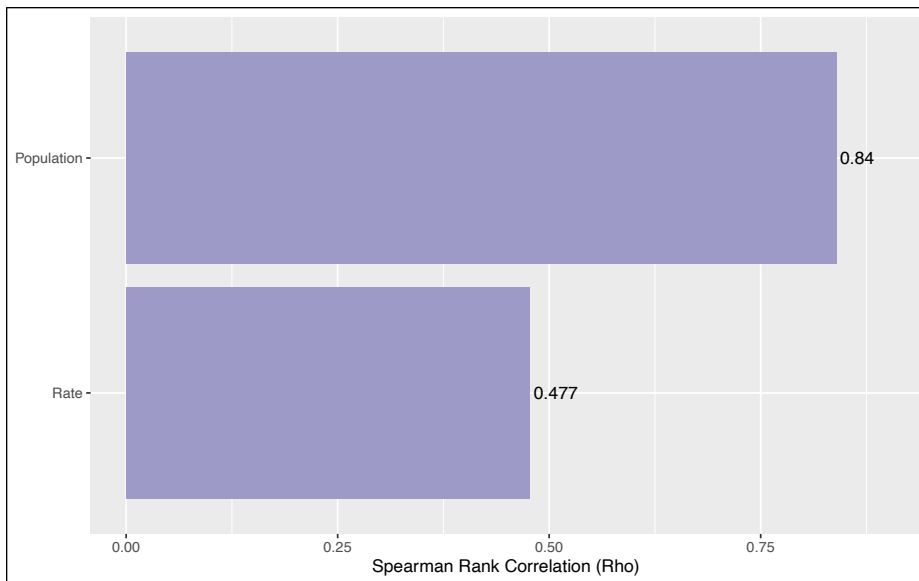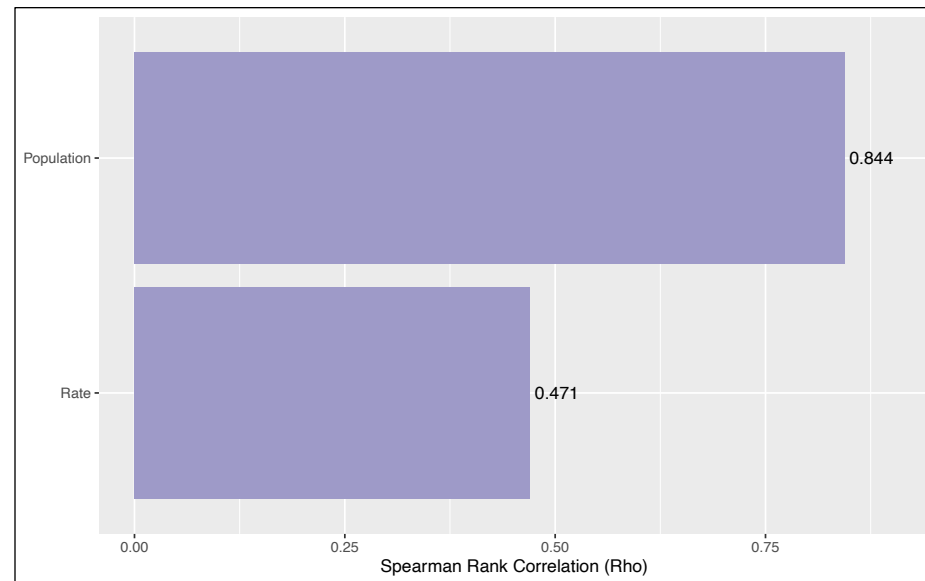

Supplement: SUPPLEMENTARY FIGURE 11 — Tornado plot for Spearman rank correlation of the output from Monte Carlo simulations to assess the sensitivity of age-standardized incidence (ASIR) and mortality (ASMR) rates by region and Comuna. [file Supplementary_Image_11.pdf]

Incidence

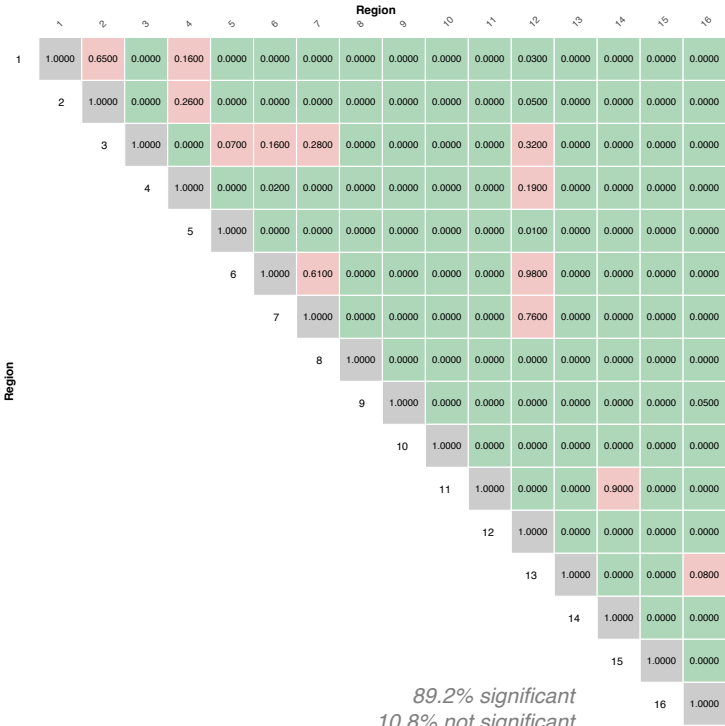

Mortality

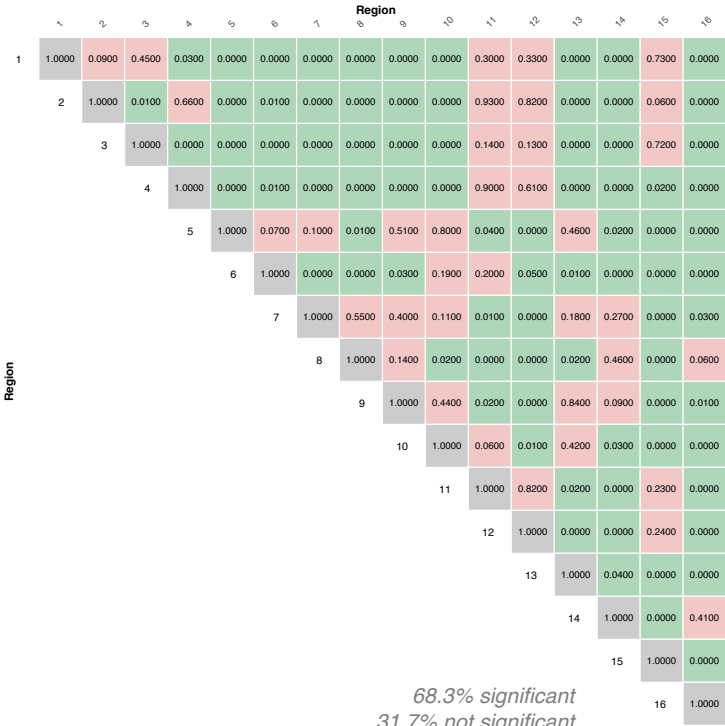

Supplement: SUPPLEMENTARY FIGURE 12 — P-value matrix heatmaps of Z-tests to assess the variance of the difference between age-standardized incidence (ASIR) and mortality (ASMR) rates by Region. [file Supplementary_Image_12.pdf]

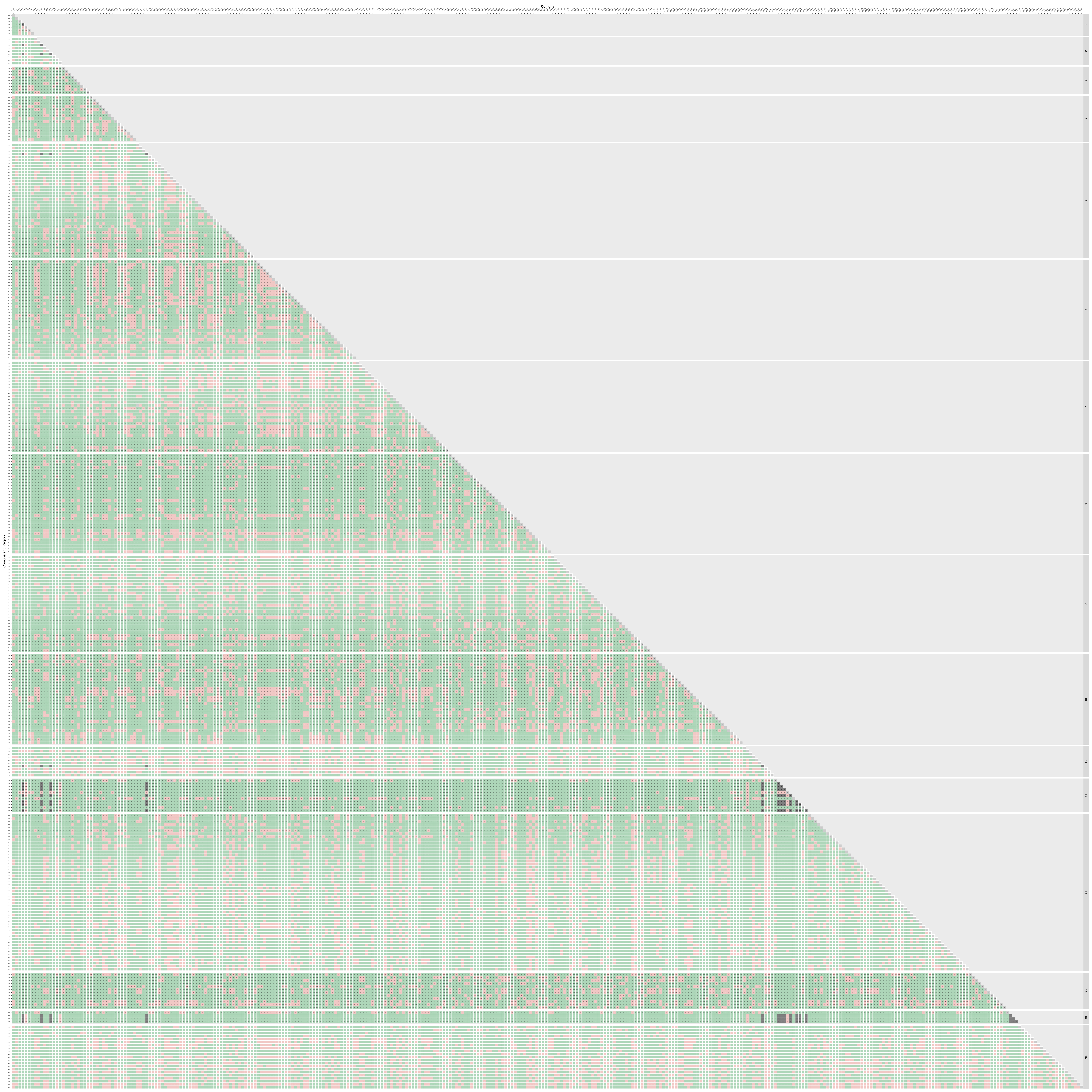

Supplement: SUPPLEMENTARY FIGURE 13 — P-value matrix heatmaps of Z-tests to assess the variance of the difference between age-standardized incidence rates (ASIR) by Comuna. [file Supplementary_Image_13.pdf]

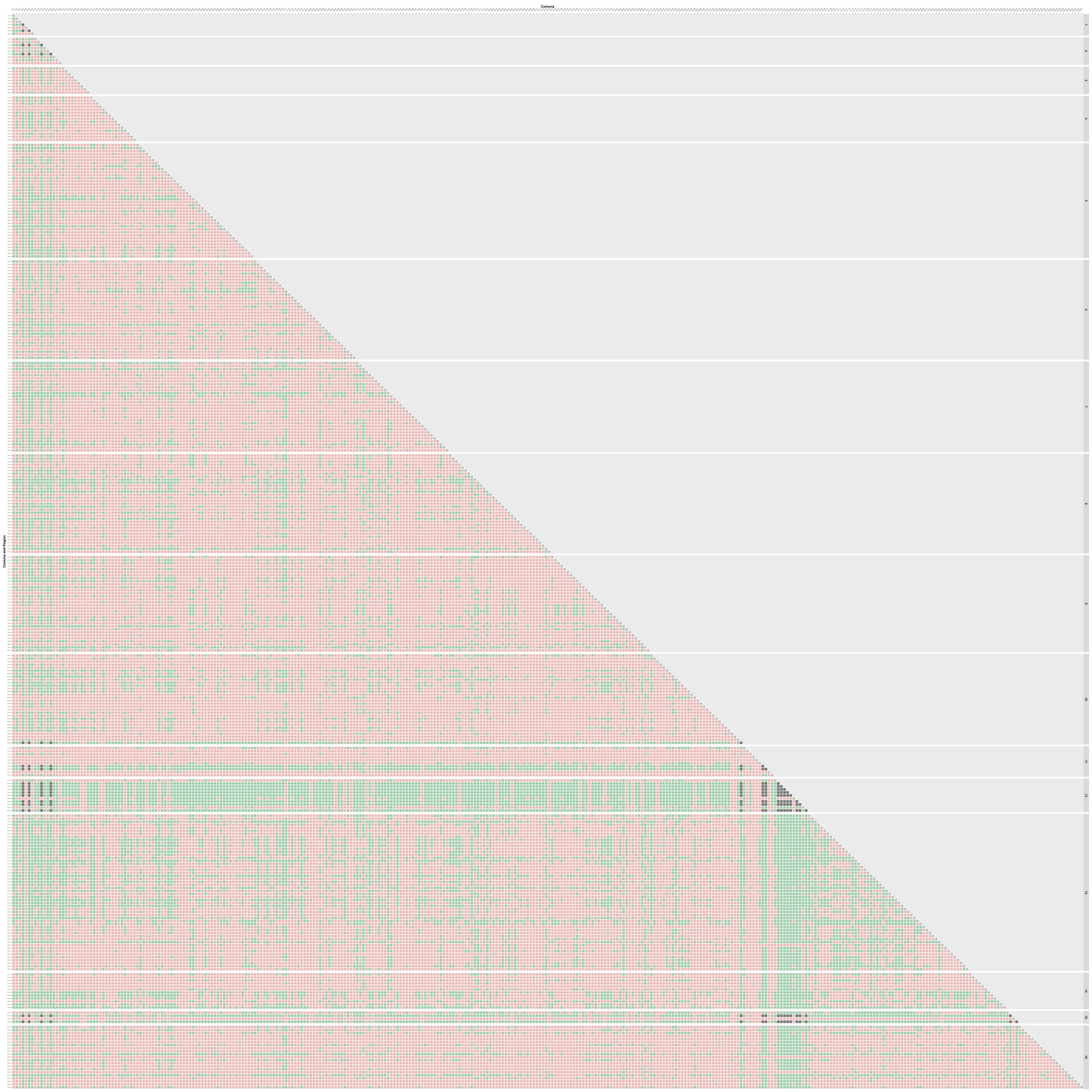

Supplement: SUPPLEMENTARY FIGURE 14 — P-value matrix heatmaps of Z-tests to assess the variance of the difference between age-standardized mortality rates (ASMR) by Comuna. [file Supplementary_Image_14.pdf]

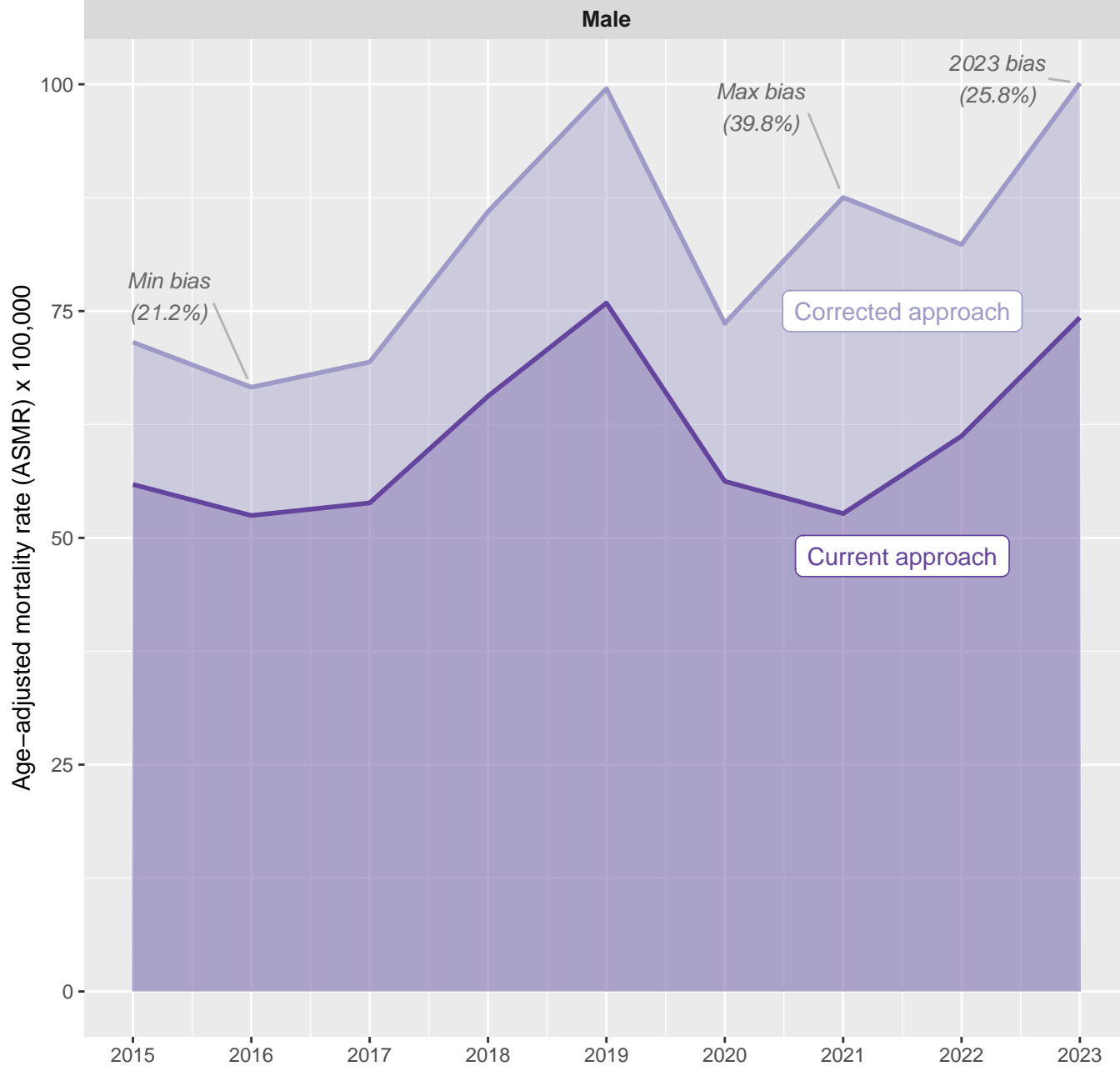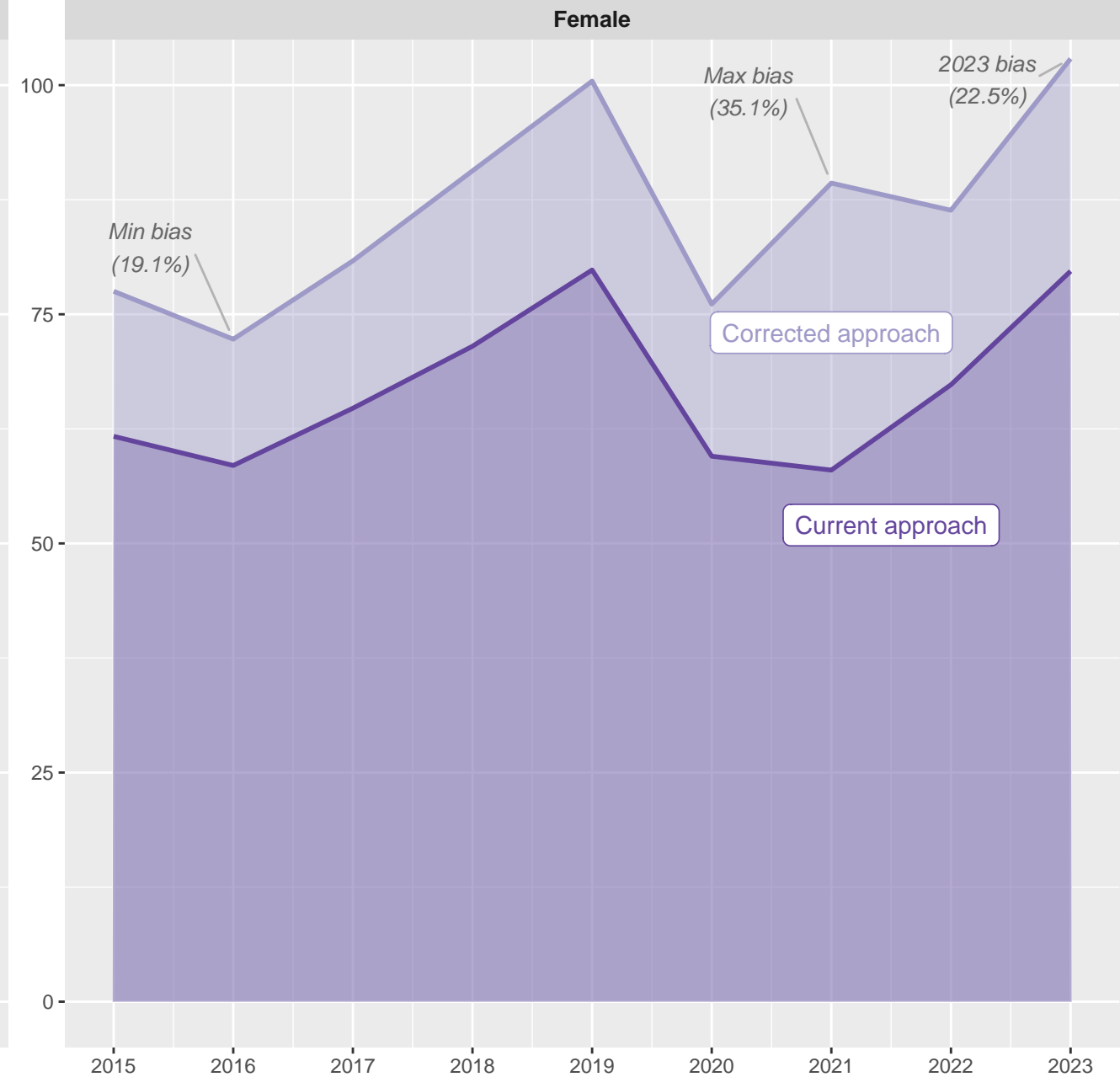

Supplement: SUPPLEMENTARY FIGURE 15 — Comparison of sepsis-related age-standardized mortality rate (ASMR) progression in Chile from 2015 to 2023 by approach to define sepsis. The current approach is based on the single-cause-of-death (SCODe) definition. The corrected approach is based on recalculating SCODe using a correction factor λ derived from representative external data. [file Supplementary_Image_15.pdf]
