# Supplementary material for: A data science-led strategy to assess the subnational burden of sepsis using official records: a longitudinal description and cross-sectional demonstration in Chile
Source: Front Med (Lausanne). 2026 Jan 12;12:1671206. doi: 10.3389/fmed.2025.1671206 (PMC12832715; doi:10.3389/fmed.2025.1671206)
Supplement: SUPPLEMENTARY FIGURE 2 — Algorithm of inclusion/exclusion for Chilean hospital discharge datasets used in this study. [file Supplementary_Image_2.pdf]

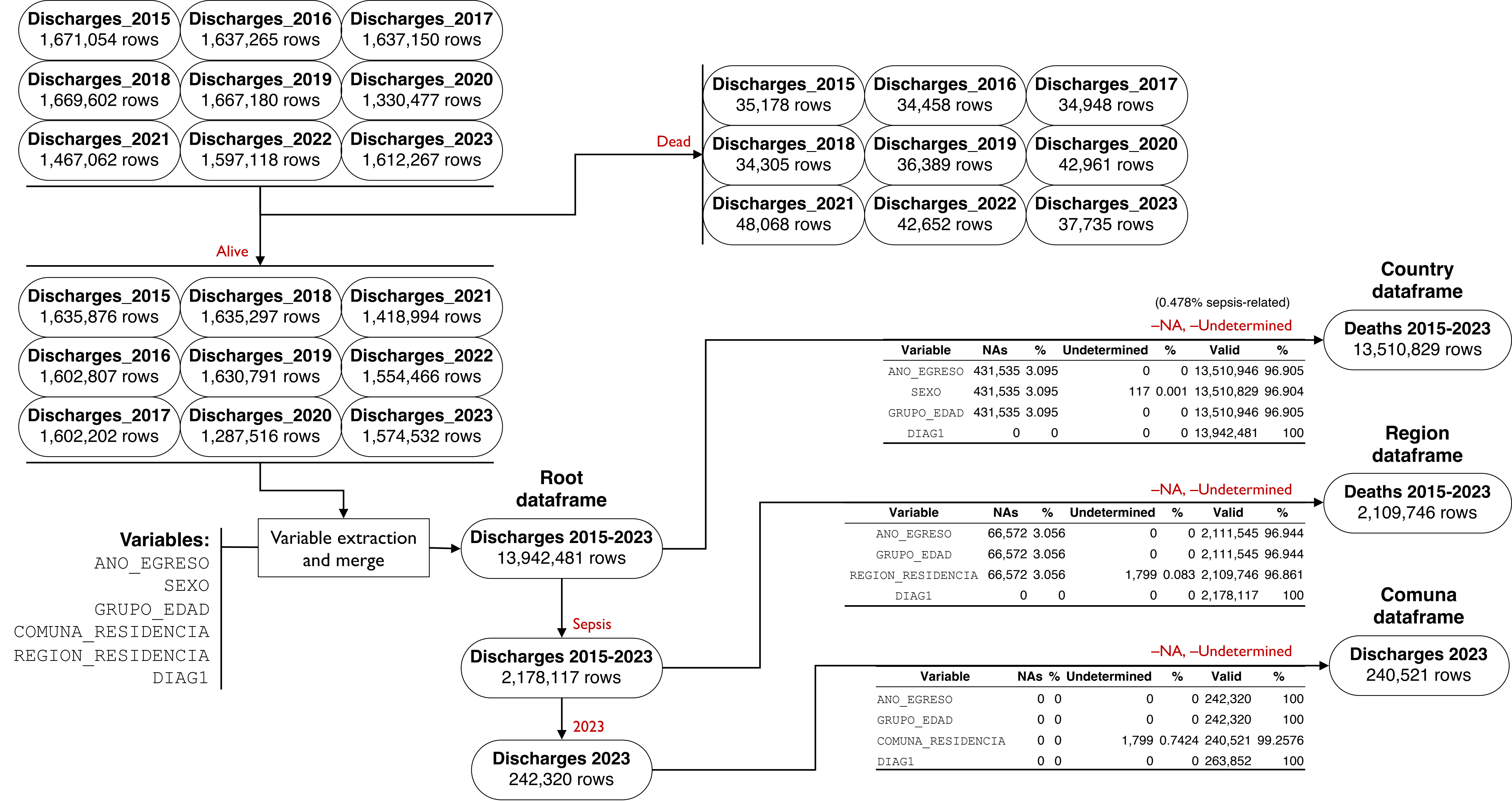

Variable	NAs	%	Undetermined	%	Valid	%
ANO_EGRESO	431,535	3.095	0	0	13,510,946	96.905
SEXO	431,535	3.095	117	0.001	13,510,829	96.904
GRUPO_EDAD	431,535	3.095	0	0	13,510,946	96.905
DIAG1	0	0	0	0	13,942,481	100

Variable	NAs	%	Undetermined	%	Valid	%
ANO_EGRESO	66,572	3.056	0	0	2,111,545	96.944
GRUPO_EDAD	66,572	3.056	0	0	2,111,545	96.944
REGION_RESIDENCIA	66,572	3.056	1,799	0.083	2,109,746	96.861
DIAG1	0	0	0	0	2,178,117	100

Variable	NAs	%	Undetermined	%	Valid	%
ANO_EGRESO	0	0	0	0	242,320	100
GRUPO_EDAD	0	0	0	0	242,320	100
COMUNA_RESIDENCIA	0	0	1,799	0.7424	240,521	99.2576
DIAG1	0	0	0	0	263,852	100
