# Supplementary material for: A data science-led strategy to assess the subnational burden of sepsis using official records: a longitudinal description and cross-sectional demonstration in Chile
Source: Front Med (Lausanne). 2026 Jan 12;12:1671206. doi: 10.3389/fmed.2025.1671206 (PMC12832715; doi:10.3389/fmed.2025.1671206)
Supplement: SUPPLEMENTARY FIGURE 5 — Definition of sepsis based on single (SCOD) or multiple cause of death/discharge (MCOD) approaches (A). Illustration of external data (Brazil death datasets) structure and application of SCOD/MCOD (B). [file Supplementary_Image_5.pdf]

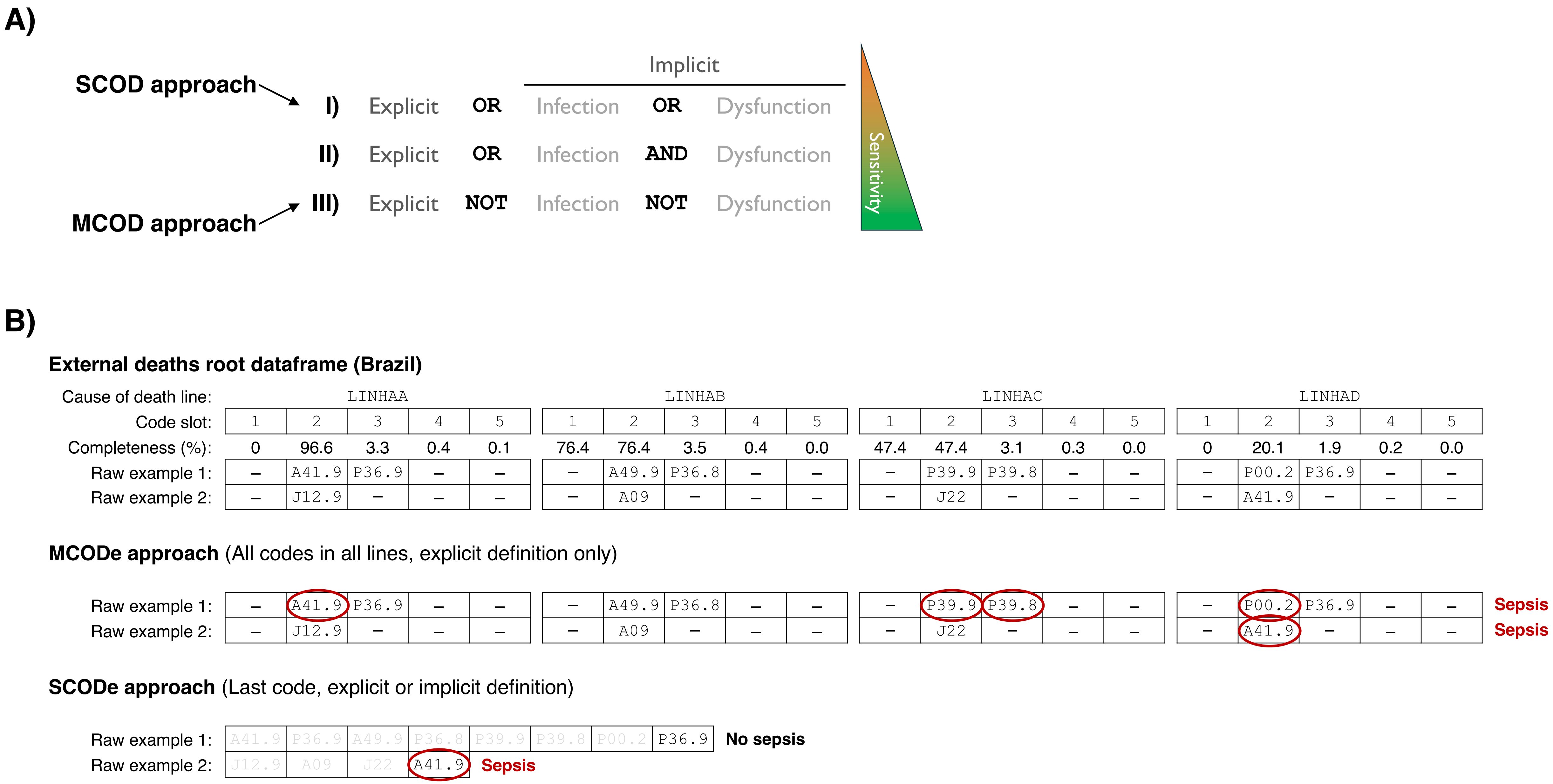
A)
SCOD approach
MCOD approach
Implicit
I) Explicit OR Infection OR Dysfunction
II) Explicit OR Infection AND Dysfunction
III) Explicit NOT Infection NOT Dysfunction
Sensitivity
B)
External deaths root dataframe (Brazil)
Cause of death line:
LINHAA
LINHAB
LINHAC
LINHAD
Code slot:
1 2 3 4 5
Completeness (%):
0 96.6 3.3 0.4 0.1
76.4 76.4 3.5 0.4 0.0
47.4 47.4 3.1 0.3 0.0
0 20.1 1.9 0.2 0.0
Raw example 1:
– A41.9 P36.9 – –
– A49.9 P36.8 – –
– P39.9 P39.8 – –
– P00.2 P36.9 – –
Raw example 2:
– J12.9 – – –
– A09 – – –
– J22 – – –
– A41.9 – – –
MCODe approach (All codes in all lines, explicit definition only)
Raw example 1:
Raw example 2:
Sepsis
Sepsis
SCODe approach (Last code, explicit or implicit definition)
Raw example 1:
A41.9 P36.9 A49.9 P36.8 P39.9 P39.8 P00.2 P36.9
No sepsis
Raw example 2:
J12.9 A09 J22 A41.9
Sepsis
