# Supplementary material for: A data science-led strategy to assess the subnational burden of sepsis using official records: a longitudinal description and cross-sectional demonstration in Chile
Source: Front Med (Lausanne). 2026 Jan 12;12:1671206. doi: 10.3389/fmed.2025.1671206 (PMC12832715; doi:10.3389/fmed.2025.1671206)
Supplement: SUPPLEMENTARY FIGURE 10 — Geographic distribution of sepsis-related age-standardized incidence rate (ASIR), age-standardized mortality rate (ASMR), and excess of mortality (EM), during 2023 by age. [file Supplementary_Image_10.pdf]

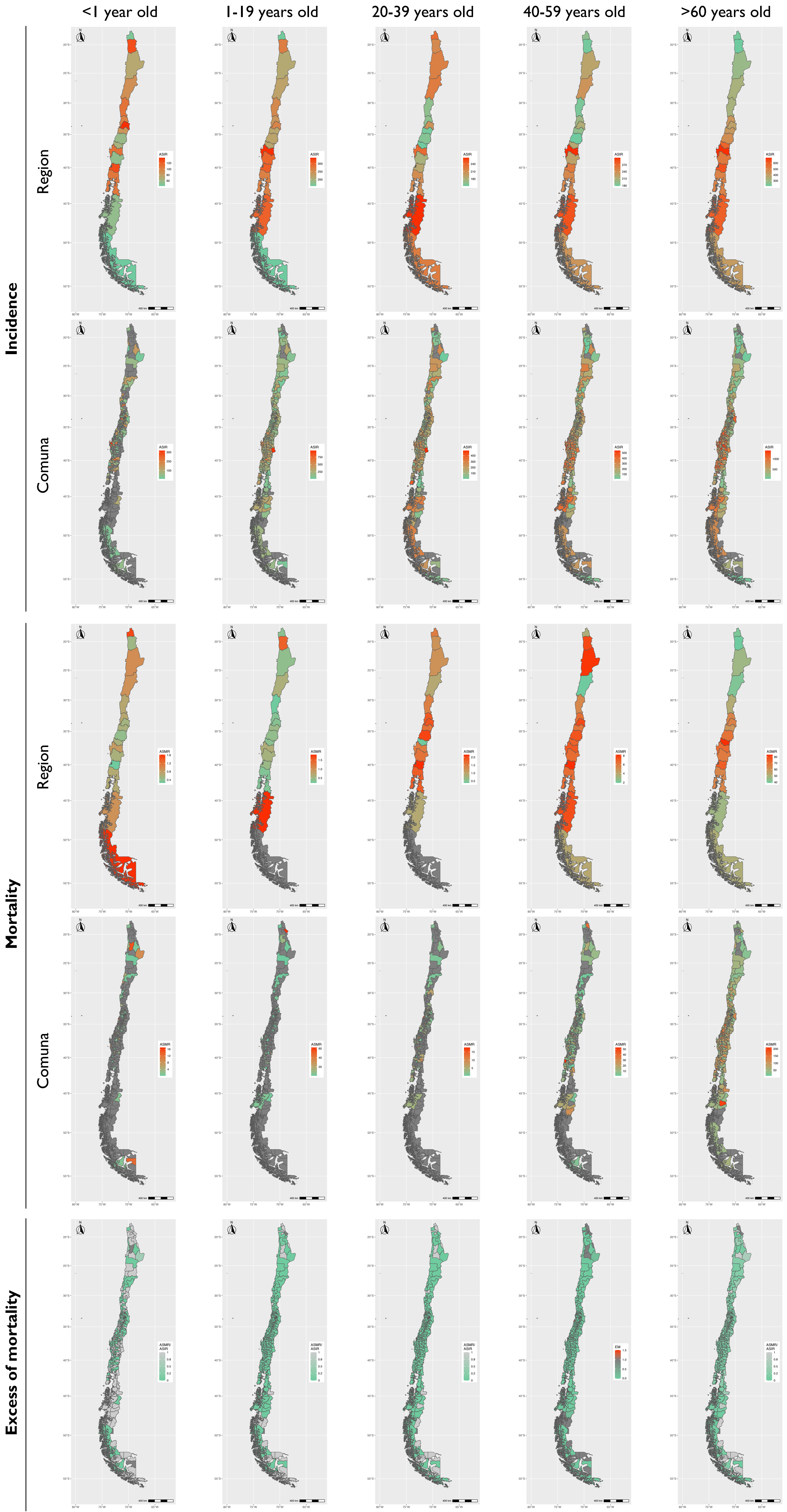

<1 year old
1-19 years old
20-39 years old
40-59 years old
>60 years old
Incidence
Region
Comuna
Mortality
Region
Comuna
Excess of mortality
ASIR
ASMR
ASMR/ASIR
EM
