# Supplementary material for: A data science-led strategy to assess the subnational burden of sepsis using official records: a longitudinal description and cross-sectional demonstration in Chile
Source: Front Med (Lausanne). 2026 Jan 12;12:1671206. doi: 10.3389/fmed.2025.1671206 (PMC12832715; doi:10.3389/fmed.2025.1671206)
Supplement: SUPPLEMENTARY TABLE 1 — Type of coding for age in the root dataframes of death, discharge, and population. [file Supplementary_Table_1.pdf]

Database	Age expressed as	Categories or magnitudes
Population	Categorical variable	0, 1, 2, 3, 4, 5, 6, 7, 8, 9, 10, 11, 12, 13, 14, 15, 16, 17, 18, 19, 20, 21, 22, 23, 24, 25, 26, 27, 28, 29, 30, 31, 32, 33, 34, 35, 36, 37, 38, 39, 40, 41, 42, 43, 44, 45, 46, 47, 48, 49, 50, 51, 52, 53, 54, 55, 56, 57, 58, 59, 60, 61, 62, 63, 64, 65, 66, 67, 68, 69, 70, 71, 72, 73, 74, 75, 76, 77, 78, 79, 80
Deaths	Continuous variable	0, 1, 2, 3, 4, 5, 6, 7, 8, 9, 10, 11, 12, 13, 14, 15, 16, 17, 18, 19, 20, 21, 22, 23, 24, 25, 26, 27, 28, 29, 30, 31, 32, 33, 34, 35, 36, 37, 38, 39, 40, 41, 42, 43, 44, 45, 46, 47, 48, 49, 50, 51, 52, 53, 54, 55, 56, 57, 58, 59, 60, 61, 62, 63, 64, 65, 66, 67, 68, 69, 70, 71, 72, 73, 74, 75, 76, 77, 78, 79, 80, 81, 82, 83, 84, 85, 86, 87, 88, 89, 90, 91, 92, 93, 94, 95, 96, 97, 98, 99, 100, 101, 102, 103, 104, 105, 106, 107, 108, 109, 110, 111, 112, 113, 114, 115, 116, 117, 118, 121, 123, 126
Discharges	Categorical variable	0, 01-09, 10-19, 20-29, 30-39, 40-49, 50-59, 60-69, 70-79, 80+
