# Supplementary material for: A data science-led strategy to assess the subnational burden of sepsis using official records: a longitudinal description and cross-sectional demonstration in Chile
Source: Front Med (Lausanne). 2026 Jan 12;12:1671206. doi: 10.3389/fmed.2025.1671206 (PMC12832715; doi:10.3389/fmed.2025.1671206)
Supplement: SUPPLEMENTARY TABLE 2 — List of ICD-10 codes used to identify sepsis in this study. [file Supplementary_Table_2.pdf]

	Implicit codes	
Explicit codes	Infection codes	Organ dysfunction
A02.1-A02.9, A20.7-A20.9, A21.7-A21.9, A22.7-A22.9, A24.1-A24.9, A26.7-A26.9, A28.2-A28.9, A32.7-A32.9, A39.0, A39.4-A41.9, A42.7-A42.9, A50-A50.9, A54.86, B00.7-B00.9, B37.7-B37.9, N98.0, O03.0, O03.38, O03.5, O03.88, O04.5, O04.88, O07.38, O08.0, O08.83, O23-O23.93, O41.1-O41.93, O75.3, O85- O86.89, O88.3-O88.32, O91- O91.23, O98, O98.2-O98.93, P00.2, P22-P23.9, P29.12, P29.81, P35-P37, P37.1-P39.9, R65.2-R65.21, R68.13	A01-A02.0, A03-A09.9, A19-A20.3, A21-A21.3, A22-A22.2, A23-A24.0, A25-A26.0, A27-A28.1, A31-A32.12, A36-A39, A39.1-A39.3, A42-A42.2, A43-A46.0, A48-A49.9, A59-A59.9, A65-A65.0, A69-A69.1, A74, A74.8-A75.9, A77-A81.9, A83-A96.9, A98-B00.59, B01-B10.89, B25-B27.99, B29.4, B33-B34.9, B37-B37.6, B38-B50.9, B54-B55, B55.1-B55.9, B58- B60.8, B64, B67-B67.99, B91, B95-B99.9, G00-G08.0, G14-G14.6, H05.01-H05.039, H60.2-H60.23, H70.0-H70.009, I00, I02, I02.9, I26.01- I26.09, I26.90-I26.99, I33-I33.9, I38-I39.9, I40.0-I40.9, I76, I96-I96.9, I98.1, J01-J06.9, J09-J22.9, J36-J36.0, J39.0-J39.1, J85-J86.9, K35- K37.9, K57-K57.93, K61-K61.4, K63.0-K63.1, K65-K65.9, K67.8, K75.0- K75.1, K75.3, K76.3, K77.0, K81.0, K81.2, K83.0, K95.01, K95.81, L02- L08.9, M00-M02.9, M86-M86.9, M89.6-M89.69, N10-N10.9, N15.1-N15.9, N30-N30.91, N39.0, N41.0, N41.2-N41.3, N45-N45.9, N70-N77.8, R78.81, T80.2-T80.29, T81.4, T82.6-T82.7, T83.5, T83.6, T84.5-T84.7, T85.7, T88.0, U04	D65-D65.9, D69.5-D69.59, E87.2-E87.99, G93.4-G93.49, I46-I46.9, I95.1-I95.9, J80- J80.9, J95.2-J95.3, J96-J96.92, K72-K72.91, N00-N01.9, N17-N17.9, R09.02, R09.2, R40.0-R40.4, R41.82, R55-R55.0, R57-R57.9
