# Supplementary material for: A data science-led strategy to assess the subnational burden of sepsis using official records: a longitudinal description and cross-sectional demonstration in Chile
Source: Front Med (Lausanne). 2026 Jan 12;12:1671206. doi: 10.3389/fmed.2025.1671206 (PMC12832715; doi:10.3389/fmed.2025.1671206)
Supplement: SUPPLEMENTARY TABLE 15 — Output of chi-square goodness-of-fit tests performed on undetermined cases in the variables age group and Comuna before exclusion from the Comuna hospital discharge dataframe (Supplementary Figure 2). [file Supplementary_Table_15.pdf]

Variable	Categories	Observed	Expected	Pearson residuals	Chi-square contribution
Age group	90 y más	29	163.55	-10.52	110
	1 a 9	277	163.55	8.87	78
	30 a 39	219	163.55	4.34	18
	10 a 19	112	163.55	-4.03	16
	80 a 89	117	163.55	-3.64	13
	menor de un año	202	163.55	3.01	9
	40 a 49	184	163.55	1.6	2
	20 a 29	161	163.55	-0.2	0
	50 a 59	165	163.55	0.11	0
	60 a 69	171	163.55	0.58	0
	70 a 79	162	163.55	-0.12	0
Comuna	88888	76	899.5	-27.46	753
	99999	1723	899.5	27.46	753
